# Population genomics of an icefish reveals mechanisms of glacier-driven adaptive radiation in Antarctic notothenioids

**DOI:** 10.1186/s12915-022-01432-x

**Published:** 2022-10-13

**Authors:** Ying Lu, Wenhao Li, Yalin Li, Wanying Zhai, Xuming Zhou, Zhichao Wu, Shouwen Jiang, Taigang Liu, Huamin Wang, Ruiqin Hu, Yan Zhou, Jun Zou, Peng Hu, Guijun Guan, Qianghua Xu, Adelino V. M. Canário, Liangbiao Chen

**Affiliations:** 1 Key Laboratory of Exploration and Utilization of Aquatic Genetic Resources (Ministry of Education), Shanghai Ocean University, Shanghai, China; 2 International Research Center for Marine Biosciences (Ministry of Science and Technology), Shanghai Ocean University, Shanghai, China; 3 Institute of Zoology, Chinese Academy of Science, Beijing, China; 4 College of Information Technology, Shanghai Ocean University, Shanghai, China; 5 Centre of Marine Sciences (CCMAR-CIMAR LA), University of Algarve, Faro, Portugal

**Keywords:** Quaternary glacial cycles, Selection sweep, Reproductive isolation, Gut microbiota, Adaptive radiation

## Abstract

**Background:**

Antarctica harbors the bulk of the species diversity of the dominant teleost fish suborder—Notothenioidei. However, the forces that shape their evolution are still under debate.

**Results:**

We sequenced the genome of an icefish, *Chionodraco hamatus*, and used population genomics and demographic modelling of sequenced genomes of 52 *C. hamatus* individuals collected mainly from two East Antarctic regions to investigate the factors driving speciation. Results revealed four icefish populations with clear reproduction separation were established 15 to 50 kya (kilo years ago) during the last glacial maxima (LGM). Selection sweeps in genes involving immune responses, cardiovascular development, and photoperception occurred differentially among the populations and were correlated with population-specific microbial communities and acquisition of distinct morphological features in the icefish taxa. Population and species-specific antifreeze glycoprotein gene expansion and glacial cycle-paced duplication/degeneration of the zona pellucida protein gene families indicated fluctuating thermal environments and periodic influence of glacial cycles on notothenioid divergence.

**Conclusions:**

We revealed a series of genomic evidence indicating differential adaptation of *C. hamatus* populations and notothenioid species divergence in the extreme and unique marine environment. We conclude that geographic separation and adaptation to heterogeneous pathogen, oxygen, and light conditions of local habitats, periodically shaped by the glacial cycles, were the key drivers propelling species diversity in Antarctica.

**Supplementary Information:**

The online version contains supplementary material available at 10.1186/s12915-022-01432-x.

## Background

The Southern Ocean (SO) has undergone a series of large episodic glacial-interglacial changes, with cycles that have lasted about 100,000 years during the latter part of the Quaternary Period (the past one million years) [1]. These environmental changes have impacted the speciation events in the SO. Through reconstructing the speciation rates across the ray-finned fishes in a geographical context, Rabosky et al. pointed out that the highest rates of marine fish speciation occurred in the high-latitude endemic fish lineages [2]. One remarkable example of such a fast speciation is the adaptive radiation of the Perciform suborder Notothenioidei. Evolved from a bottom-dwelling ancestral species in the last 40–60 million years, the notothenioids now comprise more than 120 species, with over 100 of them endemic to the high-latitude SO continental shelf waters, forming the single dominant fish taxa in this freezing environment [3].

The radiation of Antarctic notothenioids occurred contemporarily with the onset of glacial conditions in the SO [4]. Recurring glacial cycles associated with climatic oscillations are hypothesized as key ecological opportunities for the diversification of this highly endemic fauna [5]. However, during times of glacial maxima, the Antarctic nearshore habitats are periodically disrupted by grounded ice shelves and frequent ice scouring, leading to loss of habitats and decline of biodiversity [6, 7].

Much of the new insights into the mechanisms of genetic adaptation to the unique environment have been learned through the study of notothenioid genomes [8–13]. However, the factors driving the fast speciation in this clade are currently unclear. Furthermore, no population genomics studies exist and this has limited our understanding of how paleoclimatic glacial cycles impacted biodiversity.

The present study sought to investigate the role of glacial cycles in shaping current Antarctic teleost diversity through population genomics and demographic modelling of *Chionodraco hamatus*. As a member of the Channichthyidae (also known as ice fishes) family, *C. hamatus* is widely distributed in the freezing circum-high-latitude Antarctic shelf waters [14] and one of the most recently diverged notothenioids [15]. Being white-blooded due to the complete lack of hemoglobins and a near-absence of mature erythrocytes, the family Channichthyidae is perhaps the most remarkably diverged family among the eight Notothenioidei families [16] and is considered highly vulnerable in the context of coping with oceanic warming [17]. In this study, we first obtained a high-quality *C. hamatus* genome assembly, and based on which we conducted population genomics studies on individuals collected from distant East Antarctic nearshore locations. The results reveal fast population divergence and adaptive changes related to glacial cycles, yielding insights into the factors driving the radiation of the Antarctic notothenioids.

## Results

### *C. hamatus* genome assembly and population structure analysis

The high-quality assembled genome from a female *C. hamatus* has 1.15 Gb (Additional file 1: Fig. S1 and Table S1) and contains 15,016 contigs with a contig N50 at 2.18 Mb and 91.90% completeness (Additional file 1: Table S2). The genome possesses a similar GC content as the other Antarctic Notothenioids (Additional file 1: Fig. S2) and contains 50.03% repetitive sequences (Additional file 1: Table S3). A total of 806.04 Mb contigs, representing 70% of the total *C. hamatus* genome, were ordered and connected to the 24 pseudo-chromosomes of *Chaenocephalus aceratus* [9] (Additional file 1: Table S4; Suppl. Data S1). The genome was predicted to encode 30,266 proteins (Additional file 1: Fig. S3 and Table S5-7).

**Fig. 1:**
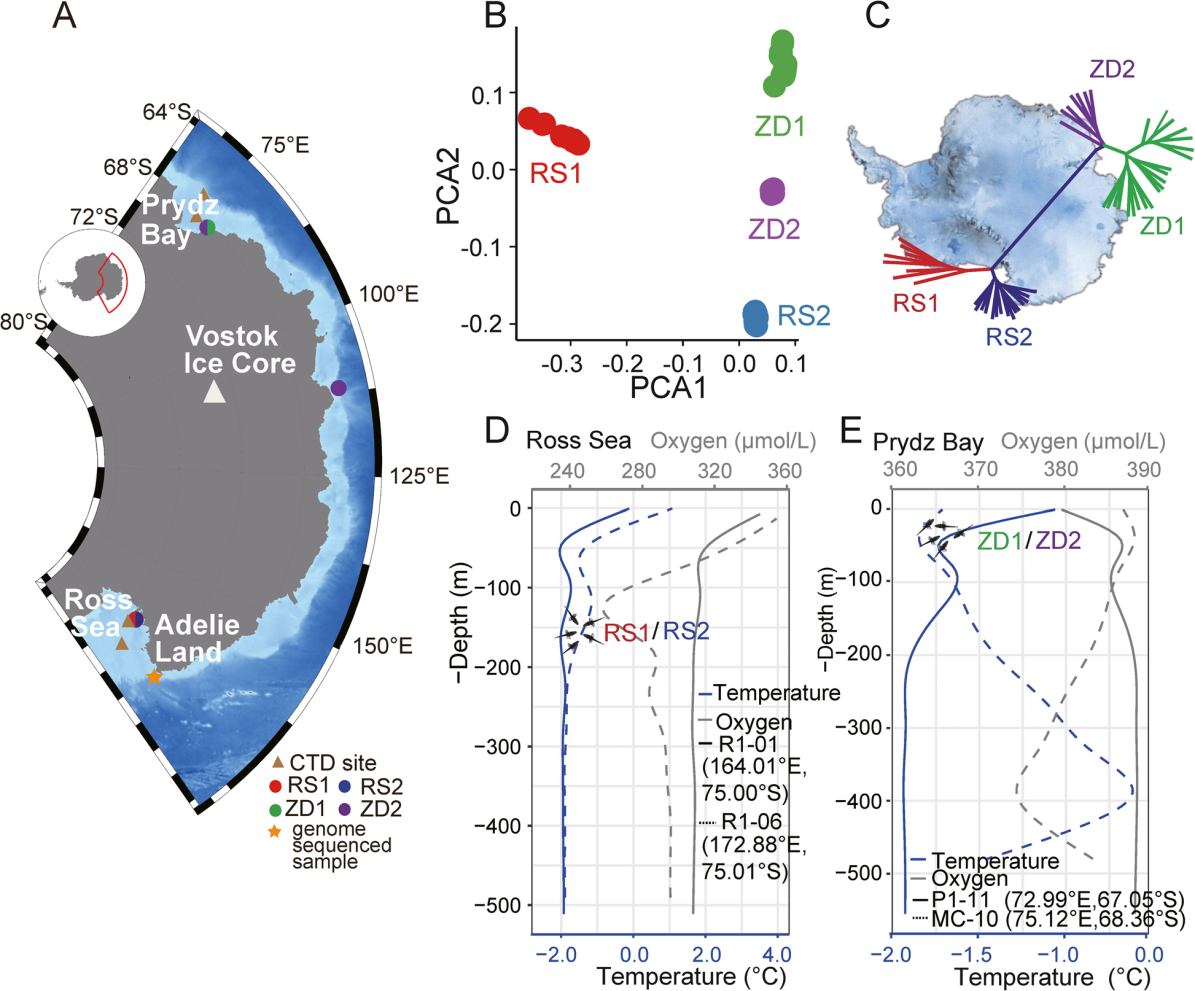
The environmental parameters of sampling sites and the *C. hamatus* population structure in the East Antarctic Continental Shelf. **A** The sampling sites (denoted by color-filled circles or semicircles for close sites) for *C. hamatus* and the CTD cast sites (yellow triangles) along the East Coast of Antarctica. **B** Principal components analysis (PCA) on SNPs from 52 *C. hamatus* individuals. RS1 (red) and another 3 populations are split by the first eigenvector (variance explained at 7.3%). RS2 (blue), ZD1 (green) and ZD2 (purple) are split by the second eigenvector (variance explained at 4.2%). **C** Maximum likelihood tree based on SNPs from the 52 individuals. The branches of each population were shown by distinct color as in **B**. The tree is positioned on top of the Antarctic map to show the two geographic locations: Ross Sea and Prydz Bay from where the majority of the samples were collected (detailed phylogenetic relationship in Additional file 1: Fig. S5). **D**,**E** The temperature-oxygen-depth curves for two Ross Sea (**D**) and two Prydz Bay (**E**) sites. Solid and dashed lines were used to distinguish two different sites of the same geographic area where CTD measurements were conducted. Blue (lower *x*-axis) and gray (upper *x*-axis) were used to denote temperature and oxygen, respectively. The data was generated by the 36th Antarctic Exploration Team of China during January 3–6 and December 4–6, 2019, using the SBE 911 plus instrument (Sea-bird Scientific, USA)

Resequencing of the genomes of 52 *C. hamatus*, collected from multiple locations along East Antarctica (Fig. 1A), resulted in 93–98% genome coverage (Additional file 1: Table S8). To verify all the 52 individuals are authentic *C. hamatus*, a phylogenetic tree based on mitochondrial DNA polymorphism were constructed (Additional file 1: Fig. S4), which support clear separation of all 52 individuals from the two most closely related congener species, *Chionodraco myersi* and *C. rastrospinosus.* By applying stringent quality control criteria, we identified a total of 11.48 million single-nucleotide polymorphisms (SNPs) (Additional file 1: Fig. S5 and Table S9; Suppl. Data S2). Principal component analysis (PCA) on the SNPs revealed four clusters, two of which, RS1 and RS2, were individuals from the Ross Sea and the other two, ZD1 and ZD2, were from the locations between Zhongshan Station and Davies Station, Pryze Bay including the one from Casey Station (Fig. 1B; Additional file 1: Tables S9). Based on the PCA and the Tracy-Widom statistics (Additional file 1: Table S10), while the RS populations were well separated from others, the ZD populations appeared very closely related. However, phylogenetic reconstruction based on the set of SNPs supported the separation of ZD1 and ZD2 (Fig. 1C; Additional file 1: Fig. S6).

The particularly lower SNP ratio detected in RS1 than the other populations is intriguing (Additional file 1: Fig. S7). One cause could be the closer phylogenetic relationship between RS1 and the individual of the reference genome as shown in Additional file 1: Fig. S7. Furthermore, by using a draft genome assembled from Whole Genome Shotgun sequencing reads derived from an individual in ZD2, the SNP ratio detected in RS1 became higher but remained to be lower than the other two populations (RS2 and ZD1) (Additional file 1: Fig. S8), suggesting a more severe bottleneck in RS1 than in the other populations could have been in play.

Analysis of environmental parameters surrounding the fishing locations showed that temperature decreased with depth and became stable and near freezing (around −1.9 °C) at a depth below 200 m at three locations, two in the Ross Sea and one at Prydz Bay, while at the other Prydz Bay station the temperature maxima was near 400 m and was higher than in other locations. Temperature variation did exist between geographic locations and between depths. Oxygen levels also varied with stations, with a pattern that either had some parallel to temperature in the Ross Sea and opposite to temperature in Prydz Bay (Fig. 1D,E; Suppl. Data S3).

### *C. hamatus* population divergence driven by glacial cycles

**Fig. 2:**
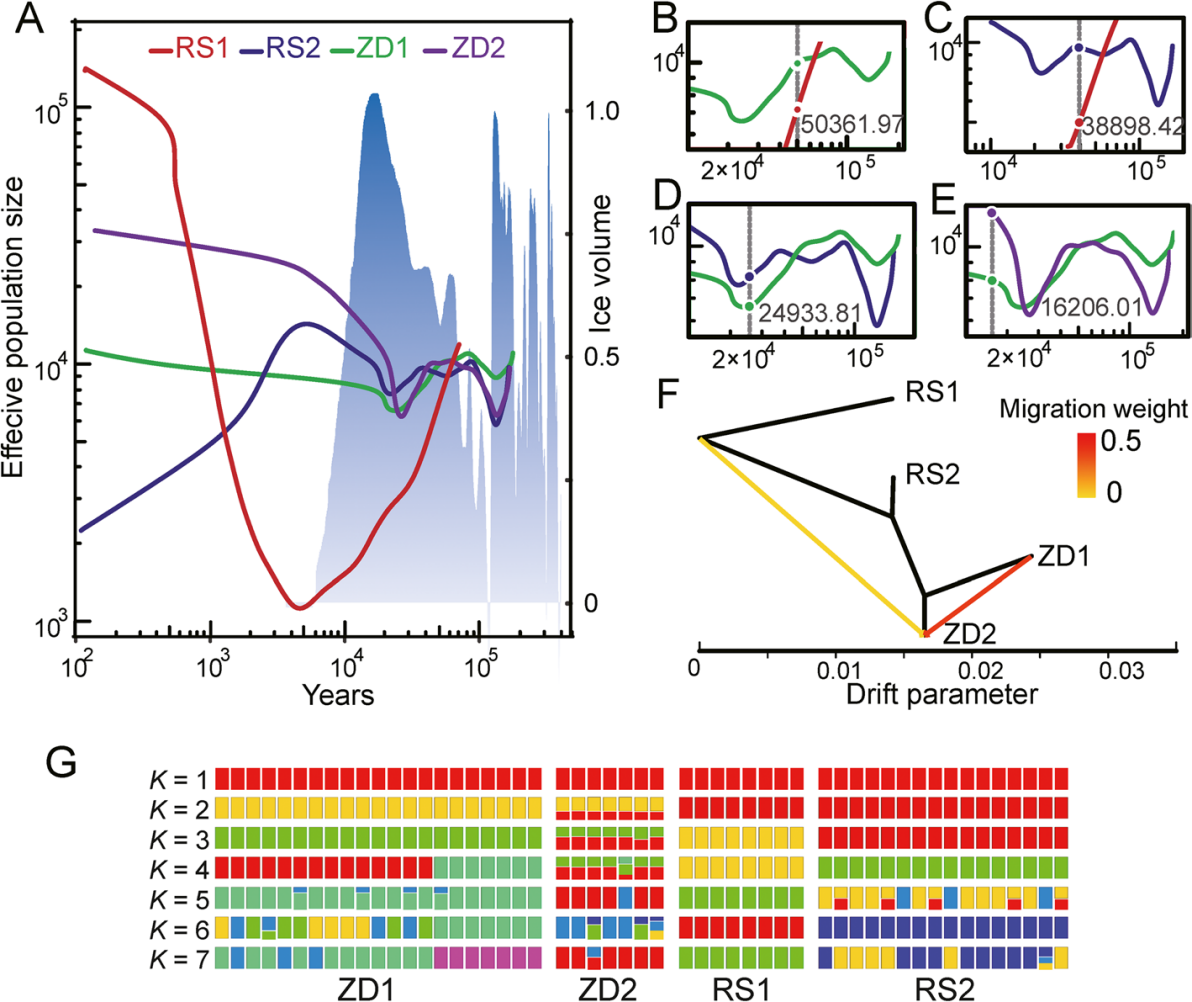
The demographic and split history of the *C. hamatus* populations. **A** Demographic history (Effective Population Size, the left *y*-axis) of the 4 populations constructed with SMC++ modelling. Generation time (*g*) is set at 7 years and neutral mutation rate per generation (*μ*) at 2.85 × 10^−8^. The blue and gray gradient represents the ice volume change (the right-side *y*-axis) over time in Antarctica, adopted from [20, 22] as a proxy of the seawater δ^18^O. The white background shows periods of lowest ice volume, corresponding to the peak interglacial warming periods. The *x*-axis represents years to present time. **B–E** Enlarged views of parts (2 × 10^4^–10^5^ years ago) of Fig. 3A to show the estimated split times between the populations. The split time is indicated by a vertical dotted line, with the corresponding number in years to the right. **B–D** show the split between RS1 (red) and ZD1 (green), RS1 (red) and RS2 (blue), RS2 (blue) and ZD1 (green) respectively, and **E** showing a more recent separation between ZD1 (green) and ZD2 (purple). **F** Maximum likelihood tree inferred by TreeMix with two migration events. Arrows are colored according to their migration weight and horizontal branch lengths are proportional to the amount of genetic drift. **G** Genetic structure of the individuals of the 4 *C. hamatus* populations inferred from ADMIXTURE by varying the ancestry components (*K*) from 1 to 7

A divergent time of 3.3 mya (1.9–4.1 mya) (million years ago) between *C. aceratus* and *C. hamatus* was obtained (Additional file 1: Fig. S9), and the neutral mutation rate calculated from Synonymous substitution rate (*dS*) / divergence time between *C. aceratus* and *C. hamatus* was 4.07 × 10^−9^ nt/year (nucleotide/year). We initially employed the pairwise sequentially Markovian coalescent (PSMC) method [18] to infer the demographic history of the *C. hamatus* populations and found signals of population fluctuation during 1 × 10^6^ to 1 × 10^4^ years ago, especially in RS1, the effective population size (*Ne*) change has been more dramatic (Additional file 1: Fig. S10). As the structuring of the *C. hamatus* populations might have occurred in a recent time frame, we then utilized SMC++ (v1.15.3) to infer the demographic history of the *C. hamatus* populations for its higher resolution in resolving the population history of the recent past [19]. We clearly simulated the demographic histories of the *C. hamatus* populations spanning a period of about 200 kya from present (Fig. 2A), which corresponded to the last two consecutive glacial cycles in the SO. Two *Ne* reductions for RS2, ZD1 and ZD2, were observed, which peaked at 120 kya and 20 kya, coinciding with the two glacial maxima of the last two glacial cycles [20–22]. It indicated an initially negative impact on the *C. hamatus* populations followed with resilient rebound when icesheets began to retreat. A shorter period of demographic history, dated back to 50 kya, was modelled for RS1, in which a continuous population decline proceeded until about 5 kya, when the ice volume reached its lowest level in the Southern Ocean, contrasting with the other populations which rebounded 20 kya (Fig. 2A). The unique demographic history of RS1 suggested an earlier clear separation of this population from the others.

Using the split function implemented in the SMC++ modeler, we established a series of separation events: 50 kya between RS1 and ZD1 (Fig. 2B), 39 kya between RS1 and RS2 (Fig. 2C) and 24 kya between RS2 and ZD1 (Fig. 2D). Although shorter, a clear separation of 16 kya between ZD1 and ZD2 was also predicted (Fig. 2E). The estimated separation time is in accordance with the unique demographic history of RS1. More interestingly, all the split events fell within the period of ice volume expansion during the latest glaciation cycle (Fig. 2A), suggesting glaciations played an essential role in the isolation of *C. hamatus* populations. Differing from a mild and uniform post-glaciation *Ne* rebounding seen in the prior cycle (about 110 kya), the current post-glaciation period witnessed mixed and scaled up *Ne* dynamics in the RS1 population. This population experienced the most severe decline during the last glaciation maxima but underwent the strongest expansion. To the contrary, RS2 reversed the post-glaciation *Ne* rebounding trend with a rapid decline contemporarily. The ZD populations, although not changing as drastically as the RS populations, showed different rates of *Ne* increase.

Given the very recent splits, we wondered whether inter-population gene flow existed, especially in populations that are geographically sympatric (i.e., RS1 and RS2, ZD1 and ZD2). By assuming different numbers of migration edges and evaluating the residual fit in TreeMix [23], we obtained the most likely migration tree with edges equal to 2 (Fig. 2F; Additional file 1: Fig. S11) and concluded that no gene flow was detected between RS1, RS2, and ZD1 within the simulated time period. The population ZD2, however, received genetic contributions from ZD1 and a historical but weaker contribution from a source closely related with the RS populations; no opposite direction of gene flow (i.e., from ZD2 to ZD1) was detected. The result indicated clear genetic separation between RS1, RS2, and ZD1, while ZD2 was not fully isolated in recent history. Population structure analysis using Admixture [24] indicated presence of ancestry components in ZD2 that were originated from ZD1 (*K* = 2) and possibly also from RS2 (*K* = 3), in addition, when *K* = 4 a distinct ancestry component not found in other populations was detected in ZD1 (Fig. 2G). These results generally agreed with the findings of the TreeMix analysis regarding ZD2. It also suggested a possible mixing of ZD1 from an unidentified source.

### The population-specific selection sweeps

**Fig. 3:**
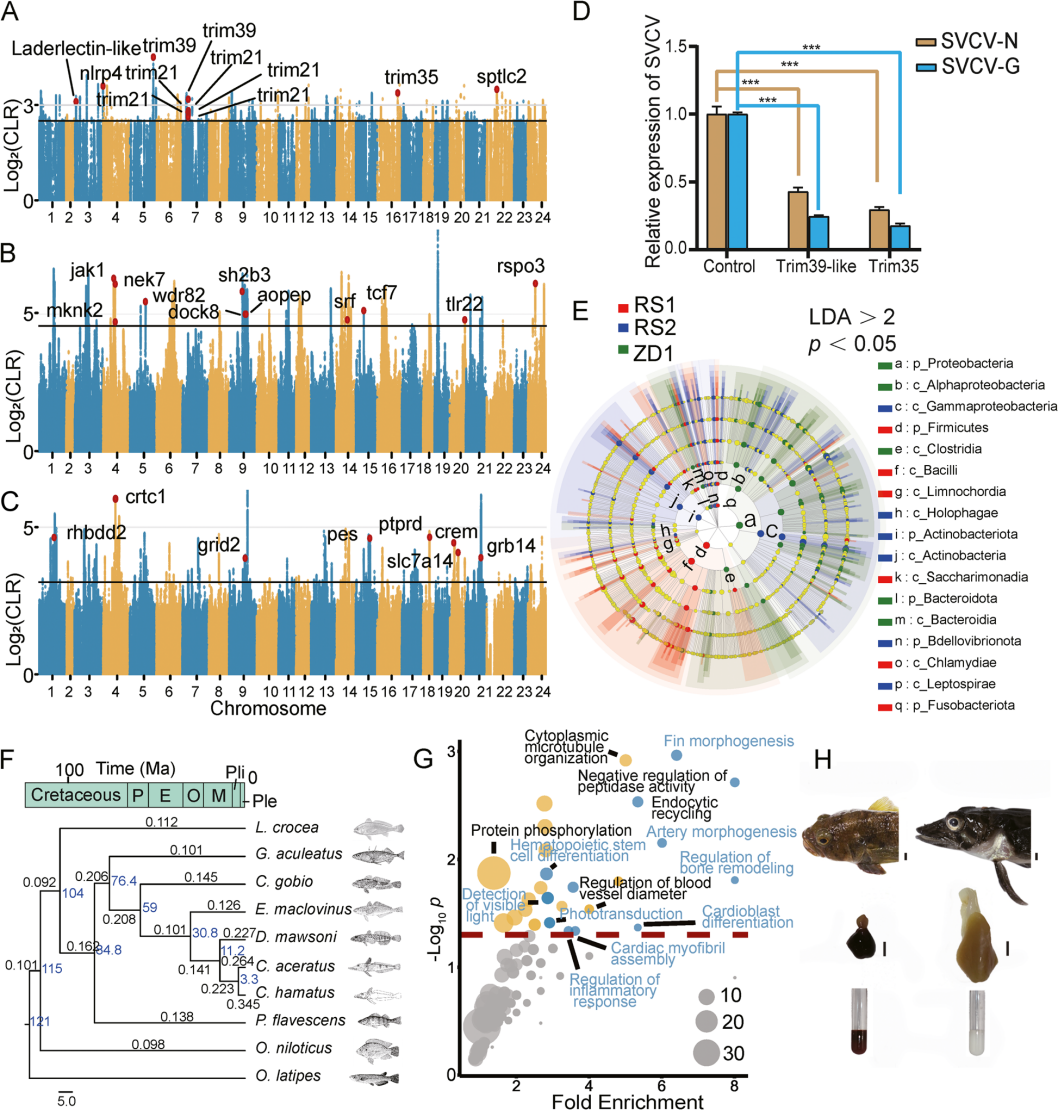
Correlation of intra-species and inter-species adaptation. **A**,**B** Manhattan plots of composite likelihood ratio (CLR) values in RS1 (**A**) and RS2 (**B**). **C** Manhattan plot of CLR values in ZD1 when matched against RS2. Genes residing within the selected regions associated with immune defense, cardiovascular development, and photoreception are labelled by gene names according to their locations. The thin solid black line in each panel denotes the top 1% strongest selective sweep regions. The numbers beneath the horizontal axis indicate the pseudo-chromosomes. **D** Inhibitory efficiencies of *trim35* and *trim39-like* on SVCV replication in transfected EPC cells measured by the relative abundance of two viral mRNAs, SVCV-N and SVCV-G. The empty vector transfected EPC cells were used as control. *** *P* < 0.001. **E** Linear discriminant analysis effect size (LEfSe) is used to classify the microbial community and assess the abundance of taxonomic units associated with each population. The six circles from inside to outside represent different taxonomic levels of the detected bacteria from phylum to species. The colored nodes represent the bacterial taxa that are significantly enriched in the population denoted by the same color (i.e., red for RS1, blue for RS2, and green for ZD1). The nodes in the light yellow indicate no significant difference in the specific taxonomic levels among the compared populations. The bacterial taxa (phylum and class) with differential abundance between the RS1, RS2, and ZD1 populations were shown to the right of the figure. The color square before the classification name denotes the same color-coded population in which the specific bacterial phylum or class is enriched (See Additional file 1: Fig. S16 and Tables S17-18 for detail information). **F** Reconstructed phylogenetic relationships of ten teleosts (“Methods”: Note 3). The black numbers above each branch refer to the *d*N/*d*S ratio. Blue numbers specify the time of divergence between species in millions of years. The geographic time is shown on the top bar. P, Paleocene; E, Eocene; O, Oligocene; M, Miocene; Pli, Pliocene; Ple, Pleistocene. **G** Significantly over-represented GO biological processes in the *C. hamatus* genes showing accelerated evolution. The blue circles represent the commonly enriched GO terms in *C. hamatus* and *C. aceratus*, while the gold circles represent the GO terms specially enriched in *C. hamatus*. The sizes of the circles represent the number of genes included in the GO term, with the scale showing in the right corner. The red dashed line indicates a *P*-value = 0.05. **H** Morphological and cardiovascular differences between *T. bernacchii* (representing red-blooded nototheniid) and *C. hamatus* (white-blooded) that correlated with the fast-evolving biological processes shown in **G**. (top) The heads of the two fishes showing drastic alterations in overall skull shape, snout length and shape, fin morphology, eye size, and presence/absence of scales. scale bar: 10 mm. (middle) A *T. bernacchii* (left) and a *C. hamatus* (right) heart. The sizes are normalized to the body lengths. Scale bar: 10 mm. (bottom) The different colors of the blood from the two fish

To see whether population-specific adaptations occurred, we identified the footprints of positive selection by pairwise comparison of the three well isolated *C. hamatus* populations (RS1, RS2, and ZD1). Firstly, the common regions with top 1% of CLR (composite likelihood ratio) values in both matchups in the three-way comparisons were identified as the selective sweep peaks for a population. Secondly, to reduce false positive genes, we polarized the SNPs of the three populations against a closely related outgroup species, *C. myersi* followed with selective sweep peak identification. The genes embedded in the common sweep peaks identified by the first and the second steps in a population were designated as the population-specific set of genes under adaptive selection. Altogether, 26 (Fig. 3A; Additional file 1: Table S11), 63 (Fig. 3B; Additional file 1: Table S12) and 49 (Fig. 3C; Additional file 1: Table S13) genes were identified respectively embedded in 18 (Additional file 1: Table S14), 49 (Additional file 1: Table S15), and 42 (Additional file 1: Table S16) selection peaks for RS1, RS2, and ZD1.

Of the 26 genes identified in the selective peaks in RS1, 11 were directly involved in immune responses (Fig. 3A). These included four *trim21* genes, *zinc finger protein RFP-like* (i.e. *trim27*), *trim35* and *trim39*, which belong to the Tripartite motif containing protein super family involved in cellular defenses against viral infections [25]. The other genes included known players in host defense and inflammation such as *NLRP4* [26], *ladderlectin-like protein* [27], and *putative serine palmitoyltransferase 2* (*spltc2*) [28].

To validate the immune-related function of the trim family genes of the icefish, we selected two genes from the largest trim subfamily contained in the *C. hamatus* genome for a functional study. Expression of the *C. hamatus trim-35* and *trim-39-like* cDNAs (Additional file 1: Table S20) [29, 30] in the Epithelioma Papulosum Cyprini cell line, drastically reduced Spring Viremia of Carp Virus (SVCV) replication (Fig. 3D; “Methods”: Note 1), indicating a conserved antiviral activity in the *C. hamatus* genes. The antiviral activity found in these two genes is similar with those found in other fishes [31, 32]. To be noticed, as many as 36 *trim35* and *trim39-like* homologous genes are present in *C. hamatus* genome (Additional file 1: Fig. S12). The widespread differential selection and expansion of the trim family genes between the populations implied a role of host defense in *C. hamatus* population divergence.

The genes under selection sweep in RS2 are also enriched in immune defense (Fig. 3B) and included *jak1* [33], *nek7* [34], *tcf7* [35], *dock8* [36], *SH2B3* [37], *tlr22* [38], *wdr82* [39], and *mknk2* [40]. These genes are involved in T cell development and maturation, interferon signal transduction, antiviral responses, and inflammatory regulation. In addition, many selection peaks in RS2 are associated with cardiac and vascular development and functioning (e.g., *serum response factor* (*srf*) [41], *aminopeptidase O-like* [42], *R-spondin-3* (*rspo3*) [43], *annexin A3* (*anxa3*) [44]). The specifically enriched positive selection genes involved in cardiovascular development in RS2 could be an evolutionary adaptation to the higher variability of the dissolved oxygen in this area, as significantly reduced levels of oxygen may occur in the Ross Sea (Fig. 1D,E). Interestingly, we observed a striking difference in the survival time in the holding tank after capture of RS1 and RS2 individuals, the former surviving much longer (Additional file 1: Fig. S13), suggesting differences in stress tolerance exist between the two populations.

Unlike the Ross Sea populations, the genes experiencing selection sweeps in the ZD1 population are associated with photoreception or retinal development (Fig. 3C): *protein eyes shut homolog* (*pes*), required to maintain the integrity of photoreceptor cells [45]; *grid2* is identified as an underlying disease gene of early-onset autosomal recessive cerebellar ataxia with retinal dystrophy [46]; *grb14* modulates rod photoreceptor response [47]; *ptprd* regulates retinal ganglion cell axon outgrowth in the developing visual system [48]; *Slc7a14*, a probable cationic amino acid transporter, and *rhbdd2*, both are linked to recessive retinitis pigmentosa [49, 50]. In addition, two genes related to photic entrainment of the circadian clock, *crtc1* [51] and *crem* [52], were also under selection. The ZD1-specific selection sweeps in genes involved in light perception might reflect the specific photic conditions the population is experiencing, since the lower latitudes and shallower depths the ZD populations currently inhabit increases exposure to light compared to the Ross Sea populations (Fig. 1D,E).

### Differential microbial communities associated with the *C. hamatus* populations

The different sets of immune-related genes under positive selection in the *C. hamatus* populations implied evolution of differential immune defenses among the populations. We characterized the gut microbial community structures of RS1, RS2, and ZD1 (“Methods”: Note 2). Of the three or four hundred bacterial species identified in each population, the majority was specifically associated with one population (Additional file 1: Fig. S14), and the microbiomes showed clear separation between the populations (Additional file 1: Fig. S15). Linear discriminant analysis (LDA score > 2, *P*-value < 0.05) identified 22 species from different phyla or classes significantly associated with RS1, 41 with RS2, and 23 with ZD1 (Fig. 3E; Additional file 1: Fig. S16, and Tables S17-18). Many of the population-specific bacteria were known pathogenic, such as mycoplasma [53] and *Acinetobacter lwoffii* [54] in RS1, *Rhodococcus erythropolis* [55] and *Mycobacterium mucogenicum* [56] in RS2, *Ralstonia pickettii* [57] and *Achromobacter* spp. [58], in ZD1, corresponding to differentiated positive selection in immune defenses between these populations.

### Correlation between intra- and interspecific selection

To determine whether the selections in the cardiovascular development, photoperception, and immune responses identified among the *C. hamatus* populations could have also been detected among notothenioid species (i.e., inter-species level), we used PAML to identify genes showing sign of accelerated evolution among 3 Antarctic notothenioids, 2 non-Antarctic notothenioids, and 5 other Percomorpha species (Fig. 3F; “Methods”: Note 3). An overall *dN*/*dS* elevation in lineages leading to the Antarctic notothenioids, especially *C. hamatus* and *C. aceratus*, was evident (Fig. 3F). Based on about 8000 well-aligned orthologous genes (“Methods”: Note 4), 945 genes in *C. hamatus*, 681 in *C. aceratus*, and 264 in *Dissostichus mawsoni* (FDR test *p* < 0.05) showed signs of accelerated evolution (Suppl. Data S4). These genes in *C. hamatus* are enriched with biological processes associated with cardiovascular development (artery morphogenesis, cardioblast differentiation, cardiac myofibril assembly) and visual perception (detection of visible light, phototransduction), together with processes such as fin morphology, bone remodelling, hemopoietic lineage differentiation, and regulation of inflammatory response (Fig. 3G). Although species differed greatly in the accelerated evolution gene sets (Suppl. Data S4), many of the biological processes represented by these genes are commonly enriched in *C. aceratus*, another white-blooded species, but are different from the red-blooded species (Additional file 1: Fig. S17). Accelerated evolution in genes associated with cardiovascular development, photoreception, immune defense, and fin regeneration was also detected in the icefish lineage when only the Notothenioid species were included for PAML analysis (Additional file 1: Fig. S18A and B). These fast-evolving biological processes are correlated with the great morphological changes occurred to the icefish (Fig. 3H) [59] which feature large heads, depressed and elongated snouts, reshaped skull, big eye, changed fin morphology, greatly enlarged hearts, and the loss of red blood cells [59].

### The glacier-paced expansion of antifreeze gene families

**Fig. 4:**
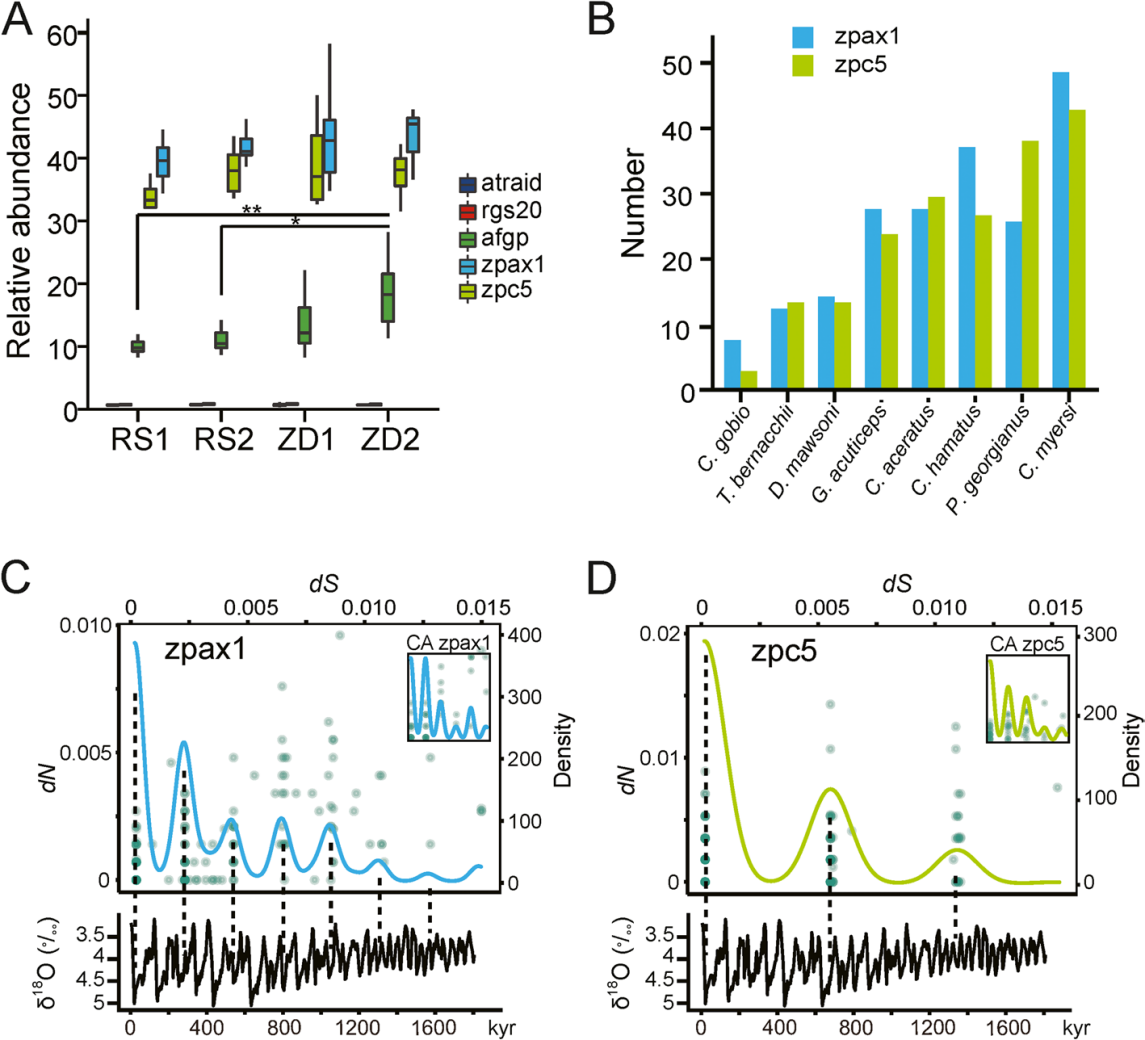
Intra-species and inter-species copy number variation (CNV) in gene families involved in freezing avoidance (**A**,**B**) and glacier-paced expansion of *ZPAX1* and *ZPC5* (**C**,**D**). **A** The relative copy number abundance of *ZPAX1*, *ZPC5*, and *AFGP* in the *C. hamatus* populations using two single-copy genes atraid, rgs20 as controls. *P < 0.05, **P < 0.01 (Kruskal-Wallis rank sum test). **B** ZPAX1 and ZPC5 CNV in notothenioid species. **C**,**D** The periodic distribution of *d*S values of the ZPAX1 (**C**) and ZPC5 (**D**) gene families. The smooth line is the *d*S density curve indicating the periodic distribution of the dS values of the two gene families in Antarctic notothenioids. The inset image in **C** and **D** is the distribution of *d*S and *d*N obtained from the *C. aceratus* ZPAX1 and ZPC5 gene sets respectively. The *d*S values were translated into the divergence time and overlayed with the δ^18^O fluctuation curve of Lisckiecki and Raymo [64]. Noting that the scales of *y*-axis in the δ^18^O curve are reversed

The notothenioids that now inhabit both the high-latitude and peripheral areas of Antarctica evolved large families of antifreeze glycoproteins (AFGPs) and zona pellucida proteins (ZPs) to prevent freezing [8, 9, 60]. The abundance of these gene families could serve as a proxy of the severity of the freezing condition a population or a species had encountered during evolution [60, 61]. We profiled the homologous sequences of *AFGPs*, *ZPAX1*, and *ZPC5* (Suppl. Data S5) in the resequencing reads of the 52 *C. hamatus* genomes and found approximately 10–18, 33–36, and 40–45 copies of the three gene families might be present in the *C. hamatus* populations. In addition, significantly more abundant AFGP coding sequences appeared to exist in ZD2 than in the other populations (Fig. 4A), seemingly correlated with an earlier population size rebounding during LGM. AFGP copy number variation (CNV) is also a feature among the notothenioid species. A recent survey in the newly sequenced notothenioid genomes further indicated the highly dynamic nature of the AFGP loci in the Antarctic species. While no AFGPs are present in the non-Antarctic *Cottus gobio*, the two Nototheniidae species (*D. mawsoni* and *Trematomus bernacchii*) contained 17 and 27 AFGP genes and another Channichthyids *Parachaenichthys georgianus* had 15 copies including 7 potentially pseudogenes [62]. The fewer copies of AFGP genes in the *C. aceratus* genome is related to a lower blood thermal hysteresis activity compared to *T. bernacchii* [63].

The zona pellucida proteins (ZPs) of the Antarctic notothenioids promote ice melt by simultaneously lowering the freezing and melting points of a solution [60]. We surveyed the copies of *ZPAX1* and *ZPC5*, the two ZP subtypes possessing the highest ice melt-promoting activities [60] in the genomes of eight notothenioids. A total of 211 *ZPAX1* and 200 *ZPC5* genes were identified (Additional file 1: Table S19; Data S6). Inter-species CNVs in the ZPAX1 and ZPC5 families are persistent. In general, icefish species possess about twice as those of the red-blooded Antarctic notothenioids (Fig. 4B).

To elucidate the relationship between ZP expansion and glacial cycles, we performed evolution analysis on *ZPAX1* and *ZPC5* gene sets from the Antarctic species (Suppl. Data S7) that were qualified for PAML analysis (“Methods”: Note 5) based on the phylogenetic trees shown in Additional file 1: Fig. S19. Remarkably, the timing of the ZP gene duplication showed strong periodicity (Fig. 4C,D). Five waves of duplication for *ZPAX1* and 3 waves for *ZPC5* with almost the same intervals were clearly identified. The *dS* differences between the serial duplication peaks were 0.0021 ± 0.00022 for *ZPAX1* (Fig. 4C) and 0.0053 ± 0.000125 for *ZPC5* (Fig. 4D). These numbers were calculated into a time (*t* = *dS* / 2μ) of approximately (2.6 ± 0.3) ×10^5^ year and (6.6 ± 1.5) ×10^5^ year, respectively, assuming the neutral mutation rate (*μ* = 4 × 10^−9^ year^−1^) of the *C. hamatus* genome. We then overlayed the timing of ZP expansion with the history of glacial cycles (represented by the fluctuating δ^18^O levels) in the past 1.8 million years [64]. The periodicities of ZPAX1 and ZPC5 expansions were approximately 3 and 6 cycles of the Quaternary glacial cycles within the past 1 mya; during this period, the SO glaciation cycled in 100 kyr. Beyond 1 mya, the ZPs expanded in the same periodicities but strode more glacial cycles due to the faster glacial cycling (about 40 kyr / cycle) (Fig. 4C,D). The phenomenon of periodic duplication of the *ZPAX1* and *ZPC5* could be detected within a single species as exhibited in *C. aceratus* with approximately the same periodicity (the inset images in Fig. 4C,D; Suppl. Data S8). These periodicities disappear if simulated *ZPAX1* and *ZPC5* sequences are applied for the analysis (“Methods”: Note 5; Additional file 1: Fig. S20). The driver for the periodic ZP gene duplication could be the need for minimal ZP dosages required to survive the periodic ice sheet advances as demonstrated by the roughly coincidence of the duplication peaks with the high δ^18^O values (proxy for low temperature) (Fig. 4C,D). Once duplicated, the redundant ZPs would inevitably be followed by a degeneration process, especially at the time of glacial retreat before triggering another round of duplication in a new glacial cycle. Copies of ZP pseudogenes were indeed spread in the genomes (the ones marked “-f” in the name in Suppl. Data S6). The faster *ZPAX1* periodicity than that of *ZPC5* might result from the different degeneration rates, as more nonsense mutations could accumulate in the bigger gene (*ZPAX1*) than in a smaller one (*ZPC5*) in a given period of time. Therefore, it appears that the strong periodicity of the Quaternary glacial cycles set the pace for duplication/degeneration of the ZP genes across the Antarctic notothenioids clade.

## Discussion

Through population genomics and comparative genomics studies, we revealed patterns of population size fluctuation in a high-latitude Antarctic (HLA) species that are near-precisely phased by the timing of glacial cycling and the periodic expansion / degeneration of gene families of the Antarctic notothenioids paced by glacial cycles, providing clear genomic evidence for a glacial cycle-driven population structuring and species divergence at least in the icefish lineage.

Our results highlight the importance of isolation and of local adaptation to conditions created by glacial cycling such as the presence of diverse pathogens, variability in oxygen concentration, and changing light conditions as the key mechanisms promoting population and species divergence in notothenioids. The common selection in the cardiovascular developmental related genes in the icefish probably resulted from the need for adequate oxygen delivery to compensate for the loss of the respiratory pigments in an environment where oxygen levels do fluctuate (Fig. 1D,E). While selective pressure for an adequate oxygen delivery could be more restricted to the hemoglobinless icefish lineage, adaptation to specific pathogens and light conditions are deemed to be more ubiquitous to the Antarctic species in general, as periodic restructuring of the nearshore biogeographic landscape by the glacial cycles frequently alter the depths and the biological composition in their habitat.

The identification of accelerated evolution in the genes involved in visual perception and phototransduction at both the population and species level pointed to light adaptation as a force shaping the icefish radiation. All icefish including *C. hamatus* can live over a large bathymetric range from the surface to a thousand meters deep where no light can reach [14]. It is likely that deep-sea niches which remained ice free during glaciation could serve as refugia for *C. hamatus* populations. Indeed, temperatures above 0°C exist at ocean depths > 1000 m (Additional file 1: Fig. S21). With the deglaciation, these deep-sea populations were able to recolonize the melted shallow benthic habitats, where light became plentily available. For the Antarctic species living broad bathymetric ranges, adaptation to light could be related to two aspects: one is the extreme daily light cycle imposed by the earth orbiting, and the second is the forced change to inhabiting depth imposed by the recurring glacial cycles.

The ubiquitously elevated evolutionary rate in the lineages leading to the individual Antarctic species and the widely spread selection sweeps within the geographically isolated populations are good indicators of local adaptations. The pronounced CNVs in the antifreeze gene families within the notothenioids suggested differential freezing conditions or different phases of glacial cycles in which the species or populations might have emerged. The glacial-phased expansion of the ZPC5 and ZPAX1 in the Antarctic notothenioids are a direct indicator for the influence of glacial cycles in the Antarctic radiation. These genomic evidence correlates well with the historical geoclimatic conditions of Antarctic that polynyas represented by the areas of permanent icesheet-free regions and the diachrony of icesheet formation during glacial maxima are widely spread in East Antarctica [65–67]. Altogether, our findings propose the important roles the glacial-driven local environmental changes had played in shaping the Antarctic radiation.

It is still uncertain whether the *C. hamatus* populations originated in situ or migrated from other geographic areas. A more complete structure of *C. hamatus* populations could be achieved when more sampling sites, especially those from the West Antarctic and the Antarctic Peninsula are included. Given the large bathymetric distribution of the species, an origin of the current populations from a deep-sea ancestral population cannot be excluded.

It is understandable that the large geographic distances between ZD1 and the Ross Sea populations, the lack of planktonic larval phase consisting instead of nesting and egg guarding, and the limited swimming capacity of icefishes [68–70] have limited gene flow. However, the mechanisms underlying the genetic separation between the two Ross Sea populations (RS1 and RS2) are so far unclear. One scenario could be geographic isolation; that is, the two populations were kept separated during the entire LGM, such that the current co-localization in the Ross Sea area is a sporadic event (e.g., a migratory path) since RS1 started its recent expansion. Indeed, we were unable to catch RS1 fishes 2 months later whereas RS2 fishes were still present in the same location. Another possibility is that the two Ross Sea populations have established genetic or behavioral barriers of reproductive isolation. A certain level of physiological differentiation might have been established between RS1 and RS2 as indicated by the disparate captive stress tolerance capabilities. The weaker stress tolerance capability of RS2 may also be a cause for its population decline when facing climatic warming and intensified ecological competition with other expanding populations, such as RS1. Need to point out that introgression could strongly affect *Ne* estimation in the sequentially Markovian coalescence-based models such as SMC++ and PSMC. Even small fractions of introgression result in waves of *Ne* increase [71]. In our cases, although PSMC curves were smooth in RS2, ZD1, ZD2 without aberrant hikings, and RS1 was on a continuous decline course during LGM, it does not rule out the existence of potential introgressions. Instead, SMC++ predicted large *Ne* increase in RS1 since 5 kya, which coincided with the warm interglacial interval when inter-species introgressions are favored [69, 72]. Whether the past and present *Ne* alterations in some *C. hamatus* populations have connections with introgression is an open question for further investigation.

The high-latitude Antarctic appears to function as an evolutionary sink for biodiversity due to the destructive effects of pronounced Pliocene-Pleistocene glacial cycles [6, 7, 73]. Negative impacts on the population sizes by glacier advance were expectedly observed in the *C. hamatus* populations. Concurrently, a clear population split without genetic flow did take place, and population-specific adaptive evolution promoted genetic divergence between the populations, which collectively increased intraspecific diversity and led to speciation. Given the quick divergence and relatively mild negative impact by glacial cycles seen in the *C. hamatus* populations, we predict that for populations that are well adapted to the HLA environment, the HLA nearshore habitats could be a hotbed for speciation in situ. Indeed, the rate of speciation with HLA origin was seen to have increased during the Quaternary glacial period [73, 74]. These same processes could also occur in the areas of Antarctic Peninsula and peripheral island archipelagos where the destructive effects of icesheets on the nearshore habitats are less intensive, making those areas historically the major source of species diversity of the Antarctic notothenioids [73].

## Conclusions

We generated a high-quality genome assembly of an HLA icefish species which is enriched with expanded transposable elements and gene families conferring viral defense and antifreeze activities. These gene families are highly dynamic between the notothenioid species and populations. We also found population-specific gut microbiota associated with the *C. hamatus* populations. Impact of glacial cycles on the evolution of the Antarctic notothenioids is reflected by the glacial cycle matched patterns of population dynamics as well as the periodic expansion and contraction of the various types of antifreeze protein gene families. The identification of differential selection sweeps in light perceptions, cardiovascular development, and immune defenses among populations suggested habitat-specific adaptation. We propose that geographic isolation followed with local adaptation has been a key mechanism for the radiation of the notothenioids in the Southern Ocean. This is also a mechanism vulnerable to the escalating global warming that may reverse the course of Antarctic radiation.

## Methods

### Fish samples and genome sequencing

The *C. hamatus* specimens were collected from East Antarctica (Fig. 1A; Additional file 1: Fig. S1) during austral summers of 2010–2011, 2015–2016, 2017–2018 by line fishing from the deck of RV XueLong with a fishing permit issued by the Office of Polar Exploration of China. Two sites were located between Zhongshan Station and Davies Station of Prydz Bay (68° 34′ 42″ S, 77° 53′ 6″ E, and 68° 33′ 40″ S, 77° 58′ E), and a third was located near the 5th Chinese Station in the Ross Sea (74° 55′ S, 163° 46′ E). A single specimen collected at the Casey station (66° 16′ S, 110° 28′ E) was also included in this study. Notably, the samples from Ross Sea and Prydz Bay were taken at different depths, i.e., approximately 130 m for the Ross Sea samples and 20–30 m for the Prydz Bay samples. The environmental parameters surrounding the fishing localities were obtained from the Conductivity, Temperature, Depth (CTD) data that casted at the four locations in the Ross Sea and in Prydz Bay, near the fishing sites.

The fishes were carefully dehooked and kept in well-oxygenated seawater for up to 5 days. They were killed with an overdose of anesthetic (MS-222), and the tissues dissected and stored at −80 °C. Genomic DNA was extracted from skeletal muscle using the Chromium^TM^ Genome Reagent Kit protocol and Genomic Tip 100/G protocol for Illumina HiSeq X Ten and PacBio SMRT-Seq sequencing, respectively. For de novo genome assembly, DNA from a female *C. hamatus* was sequenced at 87× depth coverage with PacBio and 180× coverage with Illumina shotgun reads. After filtering out the adapter sequences, low-quality reads, and duplicate reads, a total of 208.81 Gb of high-quality data were used for genome assembly. The genomes of an additional 52 individuals were sequenced at an average coverage of 13× and generating a total of 783.72 Gb. The resequencing libraries were prepared using the standard PE150 protocol and sequenced by a Novaseq 6000 (Novogene, China).

### Genome assembly

WTDBG (https://github.com/ruanjue/wtdbg) was employed to assemble PacBio long reads. Genome sequence polishing steps were applied to further improve the accuracy of the assembly. Pbalign v0.3.0 [75] was firstly used for quiver error correction, and the Illumina-generated short reads were mapped to the error-corrected assembly using the BWA v0.7.16a [76], followed with polishing by Pilon v1.21 [77]. BUSCO v5.2.2 [78] was used to evaluate genome completion using the 4584-Actinopterygii gene dataset. For genome size estimation, k-mer analysis was performed using Jellyfish v2.2.3 [79].

### Transcriptome sequencing

Total RNA was extracted from brain, duodenum, eye, fin, gill, ovary, head kidney, heart, intestine, liver, muscle, skin, and stomach using the RNAeasy Plus Mini kit (Qiagen, Valencia, CA, USA) according to the manufacturer’s instructions. PolyA+ mRNA was enriched and fragmented to synthesize cDNA using the TruSeq Stranded mRNA Library Prep Kit (Illumina, San Diego, USA). A total of 61.27 Gb sequencing data was generated on an Illumina HiSeq X Ten platform. The RNA-seq data was assembled with Trinity v2.4.0 [80] and Cufflinks v2.2.1 [81], which were integrated using PASA v2.3.3 [82].

### Genome annotation

Transposable elements were identified using RepeatModeler v1.0.11 and RepeatMasker v4.0.7 [83]. The Gene models were predicted by EvidenceModeler v1.1.0 [84], which allows the combination of predictions from ab initio, homology, and transcriptome methods. The ab initio prediction was carried out using Augustus v3.1 [85] and SNAP v2006-07-28 [86]. For the homology-based prediction, the homologous sequences of *C. aceratus* and *D. mawsoni* were aligned to the assemblies with Exonerate v2.2.0 [87].

### Identification of synteny and pseudo-chromosomal arrangement

MCScanX [88] with parameter “-s” was used to find collinear regions among *C. hamatus*, *C. aceratus*, and *D. mawsoni*. The *C. hamatus* contigs were anchored to the *C. aceratus* linkage groups according to the collinear gene blocks. A total of 806.04 Mb contigs, representing 70% of the genome, were ordered based on 24 *C. aceratus* pseudo-chromosomes [9], where the gaps between adjacent contigs were set as 100 Ns (Suppl. Data S1).

### Phylogenetic tree reconstruction and divergence time estimation

The protein-coding sequences of 10 teleost fishes were downloaded from public resources (“Methods”: Note 3; Additional file 1 Table S20). The one-to-one orthologous genes among the 10 species were identified through identification of conserved synteny and by reciprocal best hit for genes without conserved synteny. The protein sequences were aligned by PRANK [89], ambiguous regions removed by Gblocksn [90]. ProtTest [91] was used to find the best-fit model to be “JTT+I+G+F”. Phylogenetic tree was reconstructed by RAxML (with parameter: -f a -# 1000 -m PROTGAMMAIJTTF) [92]. MCMCTree implemented in PAML v4.9h [93] was used to estimate divergence time. The ages of root (97.2–127.2 mya), the common ancestor of *D. mawsoni* and *C. aceratus* (1.63–4.09 mya), and the most recent common ancestor of *Gasterosteus aculeatus* and *C. aceratus* (61.6–85.4 mya) were adopted from Timetree (http://www.timetree.org/) and used to calibrate the divergence time between *C. aceratus* and *C. hamatus*.

### Population variant calling, phylogenetic inference

The cleaned reads of 52 individuals were aligned to 24 pseudo-chromosomes using BWA [76] (Additional file 1: Table S1). SNPs and indels were called using the GATK v4.1.2.0 [94]. SNPs were firstly filtered with criteria: (1) the lowest sequencing depth of each allele ≥ 10 and (2) the minimum distance for adjacent variant ≥ 6 bp. These filtered SNPs were used for PCA analysis [95] with GCTA,v1.93.2beta [96]. To reconstruct the phylogenetic tree among individuals, SNPs were screened by customized PERL scripts. A tree based on these SNPs was constructed using RaxML (-b 1000 -m ASC_GTRCAT).

### SNP filtration

Based on the PCA and population phylogenetic tree, the 52 individuals were divided into four populations. The SNP quality of each population were further improved by filtering using VariantFiltration implemented in GATK with parameters: QD < 2.0, MQ < 40.0, FS > 60.0, SOR > 3.0, MQRankSum < −12.5, ReadPosRankSum < −8.0, and VCFtools v0.1.13 [97] with parameter of –maf = 0.05 and --max-missing = 0.02, referencing to the high-quality genome assembled from the female *C. hamatus* reported in this study.

To assess the influence of reference genome on SNP calling, we assembled an ad hoc draft genome using WGS sequencing reads (about 200× coverage) generated from a ZD2 individual. Using this ZD2-genome as a reference, we called SNPs from each individual by the same program using the same parameters. These SNP profiles were compared to the previous ones. Noteworthy, this ad hoc genome was used only for this purpose and the researches conducted throughout this study had used the SNPs generated from the high-quality genome.

### Inferring Chionodraco spp. phylogeny by mitochondrial DNA

Mitochondrial reads from the WGS data of 52 individuals were extracted by aligning to the *C. hamatus* mitochondrial genome (Accession: NC_029737.1) [98]. The variants from each individual mitochondrion were identified using the GATK program. Variants (including SNPs and indels) were used to replace reference bases to construct a mitochondrion for each individual. We then downloaded the mitochondrial genomes of *C. rastrospinosus* (Accession: NC_039543.1) and *C. myersi* (Accession: NC_010689.1) from the Genbank (population genomics data was not available for the two species) [99, 100]. These mitochondrion sequences were used to construct a maximum likelihood phylogenetic tree (RAxML，v.8.2.12，-N 1000).

### Demographic history reconstruction and gene flow estimation

The effective population size (*Ne*) history and split time of four *C. hamatus* populations were estimated using the composite likelihood method of SMC++ with parameter “-thinning *x*”, where *x* = 1000 × ln(2 × sample number). The average generation time was set at 7 years [14] and the neutral mutation rate 4.07 × 10^−9^ (unit: nt/year) were used for modelling. TreeMix v1.13 [23] was used to infer termed migration events, possible historical splits, and mixtures between populations. A maximum likelihood tree of populations was firstly produced with parameter “-k” set at 1000 to account for linkage disequilibrium. Migration edges were evaluated based on the fraction of the variance defined in the matrix of residuals. The population structure was further analyzed by Admixture [24] with the ancestry component (*K*) set at 1 to 7.

### Selection sweep identification

Genome-wide scanning for selective sweeps was performed using SweepFinder2 (-lrg 100 or -lrbg 100) [101] under the polarization of *C. myersi* or between *C. hamatus* populations, using the spatial distribution of allele frequencies and selective constraint in the genome. The Illumina reads of *C. myersi* genome were downloaded from GenBank (SRX5016434) [102]. FastEPRR v2.0 [103] was used to estimate population recombination rate and followed by background selection estimation using calc_bkgd method in the software bkgd [104]. Genome regions with a CLR [104] greater than the top 1% of CLRs were picked as candidate selective sweep regions. Candidate regions distant less than 1000 bp were connected as one sweep region, and genes fallen in these regions were identified as genes under positive selection. Effects of SNPs on genes were annotated and predicted using SnpEff v4.3t [105].

### Gut microbiome profiling

All the individuals analyzed for the microbiome were of comparable body sizes, and the individuals from the RS1 and RS2 populations were obtained from the same location in a single day. Gut microbiota DNA was isolated from 500 mg of intestinal content and quantified by spectrophotometry and agarose gel electrophoresis. About 10 ng of DNA was used for PCR amplification of the V3–V4 region of 16S ribosomal RNA using primers 338F: 5′-ACTCCTACGGGAGGCAGCA-3′ and 806R: 5′-GGACTACHVGGGTWTCTAAT-3′. Sequencing libraries were constructed using the Nextera XT Index Kit (Illumina, San Diego, USA) and paired-end sequenced (2 × 300) on an Illumina MiSeq platform (Illumina, San Diego, USA). The taxonomic affiliation of each 16S rRNA sequence was analyzed by RDP Classifier v2.11 [106] against the Silva database (138/16S_bacteria) using a confidence threshold of 70% and clustered. Linear discriminant analysis effect size (LEfSe) [107] was employed to identify the bacterial taxa significantly associated with host at all taxonomic levels.

### Antiviral activity assays

The full-length cDNAs of *Trim35* and a *trim39-like* gene were amplified by RT-PCR from *C. hamatus* liver total RNA and Sanger sequenced. The cDNAs were cloned in an expression vector driven by a zebrafish actin promoter [60]. The resultant plasmids were transfected into cultured *Epithelioma papulosum cyprini* (EPC) cells by Turbofect (Thermo Scientific). EPC cells transfected with empty vector was served as the negative control (control group). Once the transfected cells reach 90–95% confluency, 100 μl 1 × 10^7^ TCID50/ml of the Spring Viraemia of Carp Virus (SVCV) was added, and control cells were added with equal volume of DMEM/F12. The cells were cultured at 28 °C for 24 h. Viral load of the samples was quantified by qRT-PCR using the SVCV-N and SVCV-G as target genes (“Methods”: Note 1 for detail).

### Abundance profiling of the antifreeze-related gene families

Resequencing reads of 4 populations were mapped to conserved regions of *zpax1* and *zpc5* and concatenated to antifreeze glycoprotein (AFGP) coding sequences from *C. hamatus* with BWA. Two single-copy genes, atraid and rgs20, identified by OrthoMCL [108] was used as control (Suppl. Data S5). Total mapped reads of each gene were calculated using the bamCoverage algorithm in deepTools v3.5.0 [109] with parameter ignoreDuplicates. The numbers are normalized to the length of the gene and the total sequencing reads of a population to obtain the frequency of a gene in a population.

### Identification of genes under accelerated evolution

Genomic data for all the Antarctic and non-Antarctic species used in this study are downloaded from the Genbank (Additional file 1 Table S20). The orthologous protein sequences derived from conserved synteny plus reciprocal best hits from non-syntenic regions of the selected species were aligned with PRANK and transferred into corresponding codon alignments using pal2nal v14 [110]. The divergent and ambiguously aligned blocks of multiple sequence alignments were removed by Gblocks. Sequences greater than 50% of the median length of the orthologous genes of the involved species was retained for PAML analysis. The CodeML program of the PAML package was adopted to calculate genes with accelerated evolutionary rate (“Methods”: Note 4). The Benjamini & Hochberg method was used for *P*-value adjustment, and FDR < 0.05 was the cutoff used to identify accelerated evolution. Gene Ontology enrichment analysis was performed using a custom python script and a hypergeometric distribution test. Using the same set of procedures, we identified genes with accelerated evolution rate in two phylogenetic trees. (1) In the 10 species (*Larimichthys crocea*, *G. aculeatus*, *C. gobio*, *Eleginops maclovinus*, *D. mawsoni*, *C. aceratus*, *P. flavescene*, *Oreochromis niloticus*, *Oryzias latipes*) tree including non-Notothenioid species, each one of the notothenioid species was designed as the foreground branch against the background lineages of the non-notothenioid species; (2) in the tree of 11 Notothenioid species (*C. gobio*, *E. maclovinus*, *D. mawsoni*, *T. bernacchii*, *Notothenia coriiceps*, *G. aculeatus*, *Parachaenichthys charcoti*, *P. georgianus*, *C. aceratus*, *C. myersi*, *C. hamatus*), the icefish lineage was designed as the foreground branch against the background lineages of the red-blooded notothenioid species (Additional file 1: Fig. S18).

### Evolution analysis of the zona pellucida genes

The exonic sequences of seven notothenioids *(C. aceratus*, *C. hamatus*, *P. georgianus*, *C. myersi*, *G. aculeatus*, *D. mawsoni*, *T. bernacchii*) genomes identified from tBLASTn searches of *D. mawsoni zpax1* and a *zpc5* transcripts (*e*-value < 1e−80) were extracted and spliced using GlimmerHMM [111], yielding 201 *zpax1* and 198 *zpc5* homologous genes. The genes were aligned by incorporating the codon substitution model and poorly aligned sequences were excluded, resulting in 95 *zpax1* and 128 *zpc5* genes being retained to construct the phylogenic trees. The *dS* and *dN* values of each branch was calculated using the free-ratio model of the CODEML in PAML. The same processes were applied to the *zpax1* and *zpc5* genes from *C. hamatus* (“Methods”: Note 5). The AliSim program of IQ-tree v1.6.12 [112] was used simulate sequence sets based on the 95-gene *zpax1* tree, the 128-gene *zpc5* tree, and the two *C. acaratus* zona pellucida (ZP) gene trees, respectively. The simulation was run under the MG model with nonsynonymous / synonymous (*dN* / *dS*) rate ratio set at 2.0 and unequal nucleotide frequencies at 0.2, 0.3, 0.4, and 0.1 for the nucleotide A, C, G, and T, respectively, while equal nucleotide frequencies over the three codon positions were assumed. The simulated sequence sets were used for *dN* and *dS* test for each branch using the free-ratio model as above.

## Note 1

### Antiviral activity assay for the *C. hamatus* TRIM proteins

1. Cell culture

Epithelioma papulosum cyprini (EPC) cells (ATCC: CRL-2872) were maintained in DMEM/F-12 (Gibco, 11330032) containing 10% fetal bovine serum (Gibco, 10100147) and 1% penicillin-streptomycin solution (Hyclone, SV30010). The cells were grown at 28°C, 5%CO_2_ in a humidified incubator (Eppendorf, Galaxy 170R).

(2) Extraction of total RNA and cloning of the trim35 and trim39-like cDNA

Total RNA was extracted from liver of *Chionodraco hamatus* using TRIzol reagent (Invitrogen, 15596018) and precisely quantified using NanoDrop 2000. Reverse transcription (RT) was performed using a cDNA synthesis kit (Takara, RR047A), according to the manufacturer’s instructions. The sequences of the primers for *Trim35* are: forward, 5′- ATGGCTTCCAGGTTAGAGGA-3′, and reverse, 5′- ATGGCTTCCAGGTTAGAGGA-3′. The sequences of the primers for Trim39 are: forward, 5′- ATGGCTTCCAGGTTAGAGGA-3′, and reverse, 5′- CTATGTACCGTCTGTAACCCA-3′. The PCR system included 1 μl cDNA, 1.6 μl dNTP Mixture (2.5 mM each), 4 μl 5× PrimeSTAR Buffer (Mg^2+^ Plus) (Takara, R010Q), 0.8 μl forward and reverse primer, 0.2 μl PrimeSTAR HS DNA Polymerase (2.5 U/μl), and 11.6 μl ddH_2_O. Then PCR amplification was performed for 10 s at 98°C and 15 s at 68°C and 1 min at 72°C, followed by 30 cycles. The PCR product was 978 bp and sequenced.

(3) Plasmid vector construction

For convenient product detection, a Flag-tag encoding sequence (GATTACAAGGATGACGACGATAAG) was inserted before the stop codon (TGA) to express TRIM-Flag fusion protein. Using PCR, the *Trim35* and *Trim39-like* gene product of the correct size was generated and the gel-purified. The Tol2 vector was cut with the EcoRI (NEB, R3101T) and BamHI (NEB, R3136T) restriction enzymes. The enzyme digestion products were purified and linked by T4 DNA ligase (NEB, M0202S). The recombinant plasmids were sequenced to confirm the sequences.

(4) Cell transfections and SVCV infection

The *Cyprinus carpio* epithelioma papillosum cyprini (EPC) cell line was purchased from the American Type Culture Collection (ATCC, Cat No. CRL 2872). The EPC cells were seeded at a density of 6 × 10^5^cells/ml in 12-well plates. Twenty-four hours later, the cells were transfected by TurboFect transfection reagent (Thermo Scientific, R0531) with 1 μg Tol2-Trim-Flag (or Tol2) expression plasmids. For spring viraemia of carp virus (SVCV) [113, 114] infection experiment, when adherent cells reached 90–95% confluency, the experimental group added 100 μl 1 × 10^7^ TCID50/ml SVCV virus solution, the control group was added with equal volume DMEM/F12 medium. Then the cells were placed in 5% CO_2_ and 22°C incubator for 1 h. Then, cells were washed twice with DPBS (Gibco, 14190144), and complete medium was added.

(5) Sample collection, and analysis of gene expression by real-time PCR

After incubating at 28°C for 24 h, cells were collected for total RNA extraction. RNA extraction and reverse transcription were performed as described previously. Quantitative real-time PCR (qPCR) was performed using Bio-Rad CFX96 (Bio-Rad, USA) with SYBR qPCR Master Mix (Vazyme, Q511) according to the manufacturer’s instructions. The PCR amplification reactions were performed in a total volume of 20 μl containing 100 ng of cDNA, 10 μl of SYBR qPCR Master Mix, and 0.4 μl each forward/reverse primer (10 μM). And the PCR conditions were as follows: 95°C for 5 min and then 40 cycles of 95°C for 10 s, 60°C for 20 s. Specific qPCR gene primers were designed, and the β-actin gene was used as the endogenous control. Relative quantitation of mRNA expression was calculated using the 2^−∆∆CT^ method based on triplicate technical replicates. Furthermore, for the SVCV infection assay, three independent samples were prepared as biological replicates. For statistical analysis, three independent experiments were conducted.

## Note 2

### Intestinal microbial DNA extraction, high-throughput sequencing, and bioinformatic analysis

1. Intestinal content collection and total bacterial DNA extraction

About 2000 mg intestinal content was collected by scrapping the duodenum luminal mucus. The total bacterial community of gut microbiota DNA was isolated using a FastDNA® SPIN Kit (MP Biomedicals, USA) using the protocol for isolation of PCR-ready genomic deoxyribonucleic acid (DNA) from intestinal content for microbial detection designed for use with the FastPrep® Instruments from MP Biomedicals; all cells present in fecal samples are easily lysed within 40 s. DNA yield and quality were measured by NANODROP 2000 Spectrophotometer (Thermo Scientific, Wilmington, DE, USA) and agarose gel electrophoresis. The extracted genomic DNA was aliquoted and stored at −20°C prior to PCR.

(2) PCR amplification and Illumina MiSeq sequencing

The V3–V4 region of the bacterial 16S ribosomal RNA gene was amplified by PCR using primers 338F: 5′-ACTCCTACGGGAGGCAGCA-3′ and 806R: 5′-GGACTACHVGGGTWTCTAAT-3′. A total of 20 μl PCR reactions were performed as follows: 4 ml of 5× FastPfu Buffer, 2 ml of 2.5 mM dNTPs, 0.8 ml of forward primer (5 mM), 0.8 ml of reverse primer (5 mM), 0.4 ml of FastPfu Polymerase, 0.2 ml of BSA, 10 ng of template DNA, and ddH_2_O. Amplifications were performed using PCR thermal cycler Bio-Rad C1000 (Bio-Rad, USA) with an initial denaturation of 5 min at 95°C, followed by 29 cycles of 30 s at 95°C, 30 s at 55°C, and 30 s at 72°C, with a final extension of 10 min at 72°C. A PCR negative control reaction in which the genomic DNA was replaced by an equivalent volume of sterile distilled water was also included. The PCR products were visualized on a 2% agarose gel and further purified using the AxyPrep DNA Gel Extraction Kit (Axygen Biosciences, Union City, CA, USA) and quantified using QuantiFluor ™ -ST (Promega, USA) according to the manufacturer’s protocol. Primers were tagged with a unique barcode for each sample to distinguish the different PCR products, then purified PCR products were pooled in equimolar and paired-end sequenced (2 × 300) on an Illumina MiSeq platform (Illumina, San Diego, USA) and subjected to Illumina-based high-throughput sequencing (Majorbio Bio-Pharm Technology, Co., Ltd., Shanghai, China) [115].

(3) Statistical and bioinformatics analysis

The raw pair-end reads from Illumina MiSeq were firstly subjected to a quality control procedure using QIIME (version 1.9.1, http://qiime.org/install/index.html) [116, 117], and the raw fastq files were quality-filtered by Trimmomatic and merged, sequence reads that had ambiguous bases, more than one mismatch to primer sequences, homopolymers > 10 bp were merged according to their overlap with mismatch no more than 2 bp, and quality scores < 20 in a 50 bp sliding window were removed. Results with a *P*-value < 0.05 were considered significantly different. The operational taxonomic units (OTUs) were clustered with 97% similarity using UPARSE (version 7.0.1090, http://www.drive5.com/uparse/) [118]. The taxonomic affiliation of each 16S rRNA sequence was analyzed by RDP Classifier (version 2.11, https://sourceforge.net/projects/rdp-classifier/) [119] algorithm against the Silva database (138/16S_bacteria) using confidence threshold of 70% [120]. OTUs abundance information was normalized using a standard of sequence number corresponding to the sample with the least sequences. Subsequent analysis of community composition according to the results of taxonomic analysis. Principal component analysis (PCoA) [121] was used for dimensionality reduction to find the difference in sample community composition, and linear discriminant analysis effect size (LEfSe) to identify the bacterial taxa (at all taxonomic levels possible) significantly associated with host [107]. All the data analysis used the free online platform of Majorbio Cloud Platform (https://cloud.majorbio.com/).

## Note 3

### Collection of data, identification of orthologous gene, and reconstruction of phylogenetic relationship

The genomes and annotation files of *G. aculeatus*, *O. niloticus*, and *O. latipes* were downloaded from the Ensembl genome browser (Additional file 1: Table S20) [122–127]. The genome files of *D. mawsoni* and *E. maclovinus* were downloaded from the official website of the Shanghai Ocean University (Additional file 1: Table S20) [128]. The genomes of *C. gobio* and *Pantala flavescens* were downloaded from the GenBank (Additional file 1: Table S20) [129, 130]. We also downloaded the genome sequence of *C. aceratus* from Korea Polar Data Center (Additional file 1: Table S20) [131]. The genome sequence of *L. crocea* was downloaded from figshare website (Additional file 1: Table S20) [128]. The one-to-one orthologous genes among the 10 species were identified through conserved synteny and reciprocal best hits (RBH). In brief, the synteny genes of ten species were screened by MCScanX software [88], and protein sequences were aligned using the DIAMOND software [132] with an *E*-value cutoff of 1e−05. The additional orthologous genes located in the non-synteny blocks were extracted through reciprocal best hit (RBH) by blastp (v2.5.0+) [133]. The protein sequences were aligned by PRANK software [89, 134]. The aligned protein sequences of a species were converged as a “supergene”. The divergent and ambiguously aligned blocks were removed using Gblocks (0.91b) software [90] in the “supergene”. ProtTest (v3.4) [91] was used to find the best-fit model of the sequence alignment, which was “JTT+I+G+F”. Phylogenetic tree was reconstructed by RAxML (v 8.2.12) [92] with parameter: -f a -# 1000 -m PROTGAMMAIJTTF with *O. latipes* set as “outgroup”. The protein alignment was then transferred to codon alignment using pal2nal (v14) [110]. The *dN* / *dS* ratio (*ω*) representing the evolutionary rate were calculated for each lineage of the tree using the free-ratio model implemented in the PAML (codeml) package [110].

## Note 4

### Identification of genes under accelerated evolution and GO enrichment test

The genes with accelerated evolution rate were assessed by CodeML program of the PAML package using branch-site model. We set the non-notothenioids *G. aculeatus*, *O. niloticus*, *O. latipes*, *L. crocea*, and *P. flavescens* as the background and one of the notothenioids, *C. hamatus*, *C. aceratus*, *D. mawsoni*, *E. maclovinus*, and *C. gobio* as the foreground branch. Orthologous protein sequences of the selected species obtained from the conserved syntenies plus those obtained by reciprocal best hit using blastp from the non-syntenic regions were aligned using PRANK software (v.140603). The protein alignment was transferred to codon alignment using pal2nal (v14) [110]. The divergent and ambiguously aligned blocks of multiple sequence alignments were deleted by Gblocks (0.91b) software with codon model. Genes with the conserved sequence length over 50% of the median length of the homologous genes from all species involved were retained for following analysis. About 8000 orthologous sequences were retained for each PAML analysis. The branch-site model of codon evolution was used with model = 2 and NSsites = 2. The adjustment of *P*-value was implemented by Benjamini & Hochberg method and the cutoff of false discovery rate (FDR) < 0.05 is used to identify the accelerated evolution. Meanwhile, the BEB methods for calculating posterior probabilities for site classes are implemented. The GO enrichment analysis was performed by custom python script, and the hypergeometric test was used to estimate significance (*P*-value < 0.05).

## Note 5

### Evolution analysis of ZP proteins

ZPAX1 and ZPC5 genomic DNAs from seven notothenioids with a tblastn e-value < 1e−80 against a ZPAX1 and a ZPC5 transcripts of *D. mawsoni* were extracted using a custom python script. The protein-coding sequences were spliced by GlimmerHMM prediction [111]. In total, 201 zpax1 and 198 zpc5 homologous genes were obtained. The multiple sequence alignment of coding sequences of ZPAX1 and ZPC5 was carried out with PRANK incorporating a codon substitution model, and sequences aligned poorly with the majority of the sequences were excluded. Ninety-five ZPAX1 and 128 ZPC5 genes were maintained and used for evolution analysis. The poorly aligned regions were removed by Gblocks while preserving the codon structure. The length of 1968 bp of ZPAX1 and 771 bp of ZPC5 conserved sequences were used to construct the phylogenic trees using IQ-TREE software. The *dS* and *dN* values of each branch was calculated using the free-ratio model of the CODEML program in PAML. To see whether ZPs within the same species also showed similar patterns of evolution, the same processes used for the multiple-species combined sets of ZPAX1 and ZPC5 genes were also applied to single species, for example *C. aceratus*. Amount of 22 (out of 29) ZPAX1 and 25 (out of 31) ZPC5 genes were included for the analysis.

## Supplementary Information

**Additional file 1: Figure S1.** An eagle’s view of a *C. hamastus*. **Figure S2.** Comparison of genome GC Content among 5 notothenioid species. **Figure S3.** Landscape of the *C. hamatus* genome at 100 Kb scale. **Figure S4.** Maximum likelihood trees reconstructed from extracted mitochondrial reads of 52 *C. hamastus* individuals together with *C. myersi* and *C. rastrospinosus*. **Figure S5.** The phylogeny of the re-sequenced 52 individuals with individual names shown supplementary to Fig. 1C. **Figure S6.** Characteristics of SNPs identified in the *C. hamatus* populations. **Figure S7.** Phylogeny tree depicts the closer relationship between RS1 and the individual of the reference genome (REF). **Figure S8.** Results of a parallel SNP call from the 52 individuals against an ad-hoc draft genome assembled from only the WGS sequencing reads from a ZD2 individual. **Figure S9.** Reconstructed phylogenetic tree of ten fishes based on the maximum likelihood method. **Figure S10.** The demographic histories of *C. hamatus* populations, RS1, RS2, ZD1, and ZD2, parallelly estimated by Pairwise sequentially Markovian coalescent (PSMC). **Figure S11.** Corresponding scaled residuals of Fig. 2F from the fit of the TreeMix model to the data. **Figure S12.** The phylogenetic tree of the trim-35 and trim-39-like homologous genes from three icefish genomes indicating gene family dynamics between the species. **Figure S13.** Differential survival time after capture by RS1 and RS2. **Figure S14.** The Venn diagram showing the total number of bacterial species identified in three populations. **Figure S15.** Principal Component Analysis showing a population-based clustering pattern of the gut microbiota in the three populations. **Figure S16.** Differentially abundant gut microbiota species specifically associated with RS1, RS2 and ZD1 as determined by LEfSe. **Figure S17.** The enriched GO list of the positive selection genes identified in *C. hamatus*, *G. aceratus* and *D. mawsoni*, supplementary to Fig. 4G. **Figure S18.** Phylogenetic analysis and gene evolution of notothenioid fish. **Figure S19.** Phylogenetic trees of the ZPAX1 and ZPC5 genes contained in the seven Antarctic species. **Figure S20.** The distribution of dS values of the simulated sequences from the ZPAX1 and ZPC5 trees. **Figure S21.** The temperature curve along the Ocean depth. **Table S1.** Statistics of sequencing data. **Table S2.** BUSCO outputs for the *C. hamatus* genome. **Table S3.** Annotated repetitive sequences. **Table S4.** Statistics of the assemblies for each pseudo-chromosome. **Table S5.** Functional classification of the protein-coding genes according to different databases. **Table S6.** Statistics of protein-coding genes predicted by various methods. **Table S7.** Summary of non-coding RNA genes in *C. hamatus* genome. **Table S8.** Collected *C. hamatus* samples and sequencing statistics. **Table S9.** SNP calling in 4 populations. **Table S10.** Tracy-Widom (TW) statistics for the first four eigenvalues form PCA analysis. **Table S11.** The RS1 specific gene list under selective sweep. **Table S12.** The RS2 specific gene list under selective sweep. **Table S13.** The ZD1 specific gene list under selective sweep. **Table S14.** List of sweep regions of RS1, using an outlier approach in RS2. **Table S15.** List of sweep regions of RS2, using an outlier approach in RS1. **Table S16.** List of sweep regions of ZD1, using an outlier approach in RS2. **Table S17.** The microbial phyla and classes specifically associated with a *C. hamatus* population. **Table S18.** The microbial organisms specifically associated with a *C. hamatus* population. **Table S19.** ZPAX1 and ZPC5 gene copy number tally in eight notothenioids. **Table S20.** Data info of genome, annotations and reads used in this study. **Data S1-S8** are available from http://ogd.shou.edu.cn/download.html.

## Data Availability

All of the Illumina short-read sequencing data of this project have been deposited at NCBI under the accession no. BioProject PRJNA664334 (https://www.ncbi.nlm.nih.gov/bioproject/ PRJNA664334) [135]. The annotation files of the *C. hamatus* genome can been downloaded from http://ogd.shou.edu.cn/download.html [136]. The info of all other species used in this study are listed in Additional file 1: Table S20.

## Abbreviations

kya: Kilo years ago
kyr: Kilo years
LGM: The last glaciation maxima
SO: Southern ocean
SNP(s): Single-nucleotide polymorphism(s)
PCA: Principal component analysis
mya: Million years ago
ds: Synonymous substitution rate
dn: Nonsynonymous substitution rate
nt: Nucleotide
PSMC: The pairwise sequentially Markovian coalescent method in software PSMC
*Ne*: Effective population size
CLR: Composite likelihood ratio
SVCV: Spring viremia of carp virus
LDA: Linear discriminant analysis
AFGP(s): Antifreeze glycoprotein(s)
ZP(s): Zona pellucida protein(s)
CNV: Copy number variation
HLA: High-latitude Antarctic
CTD: Conductivity, Temperature, Depth
EPC: *Epithelioma papulosum cyprini*
LEfSe: Linear discriminant analysis effect size
qPCR: Quantitative real-time PCR
