# Supplementary material for: Population genomics of an icefish reveals mechanisms of glacier-driven adaptive radiation in Antarctic notothenioids
Source: BMC Biol. 2022 Oct 13;20:231. doi: 10.1186/s12915-022-01432-x (PMC9560024; doi:10.1186/s12915-022-01432-x)
Supplement: Supplementary file 1 — Additional file 1: Figure S1. An eagle’s view of a C. hamastus. Figure S2. Comparison of genome GC Content among 5 notothenioid species. Figure S3. Landscape of the C. hamatus genome at 100 Kb scale. Figure S4. Maximum likelihood trees reconstructed from extracted mitochondrial reads of 52 C. hamastus individuals together with C. myersi and C. rastrospinosus. Figure S5. The phylogeny of the re-sequenced 52 individuals with individual names shown supplementary to Fig. 1C. Figure S6. Characteristics of SNPs identified in the C. hamatus populations. Figure S7. Phylogeny tree depicts the closer relationship between RS1 and the individual of the reference genome (REF). Figure S8. Results of a parallel SNP call from the 52 individuals against an ad-hoc draft genome assembled from only the WGS sequencing reads from a ZD2 individual. Figure S9. Reconstructed phylogenetic tree of ten fishes based on the maximum likelihood method. Figure S10. The demographic histories of C. hamatus populations, RS1, RS2, ZD1, and ZD2, parallelly estimated by Pairwise sequentially Markovian coalescent (PSMC). Figure S11. Corresponding scaled residuals of Fig. 2F from the fit of the TreeMix model to the data. Figure S12. The phylogenetic tree of the trim-35 and trim-39-like homologous genes from three icefish genomes indicating gene family dynamics between the species. Figure S13. Differential survival time after capture by RS1 and RS2. Figure S14. The Venn diagram showing the total number of bacterial species identified in three populations. Figure S15. Principal Component Analysis showing a population-based clustering pattern of the gut microbiota in the three populations. Figure S16. Differentially abundant gut microbiota species specifically associated with RS1, RS2 and ZD1 as determined by LEfSe. Figure S17. The enriched GO list of the positive selection genes identified in C. hamatus, G. aceratus and D. mawsoni, supplementary to Fig. 4G. Figure S18. Phylogenetic analysis and gene evolutio [file 12915_2022_1432_MOESM1_ESM.docx]

***Additional files***

**Population genomics of an icefish reveal mechanisms of glacier-driven adaptive radiation in Antarctic Notothenioids**


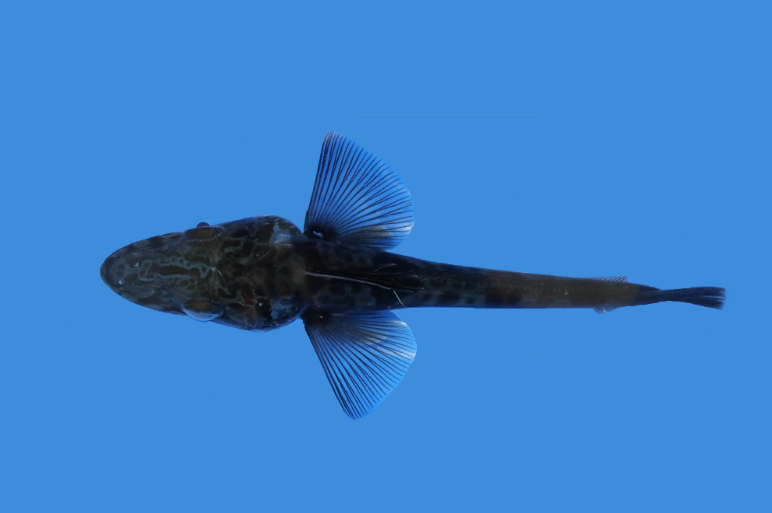


**Fig. S1**. An eagle’s view of a *C. hamatus.*


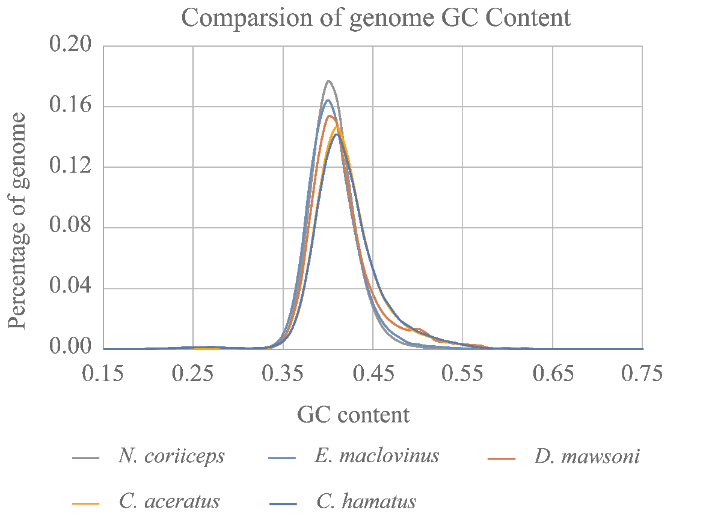


**Fig. S2.** Comparison of genome GC Content among 5 Notothenioid species.


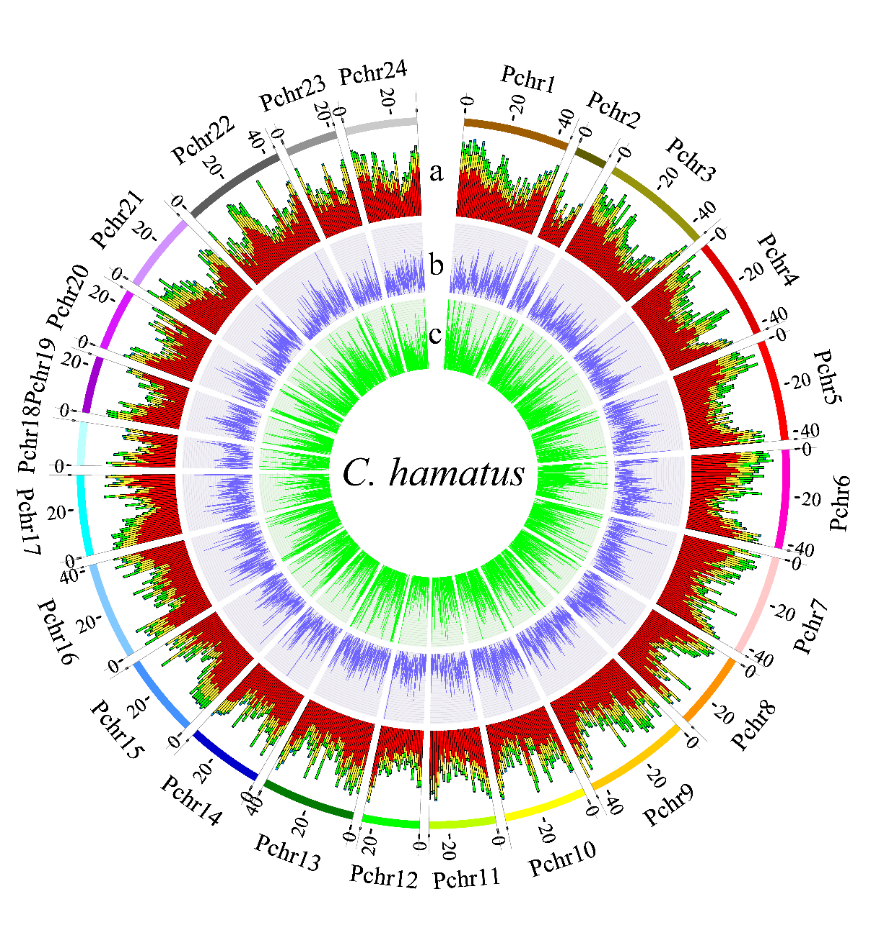


**Fig. S3.** Landscape of the *C. hamatus* genome at 100 Kb scale. From outer to inner circles: a, repetitive sequence distribution, red, green, yellow and blue represent the typical transposable elements of DNAs, LINEs, LTR and SINEs, respectively; b, density of the anchored protein-coding genes; c, distribution of GC contents across the genome. The pseudo-chromosomes are presented as Pchr1-24.


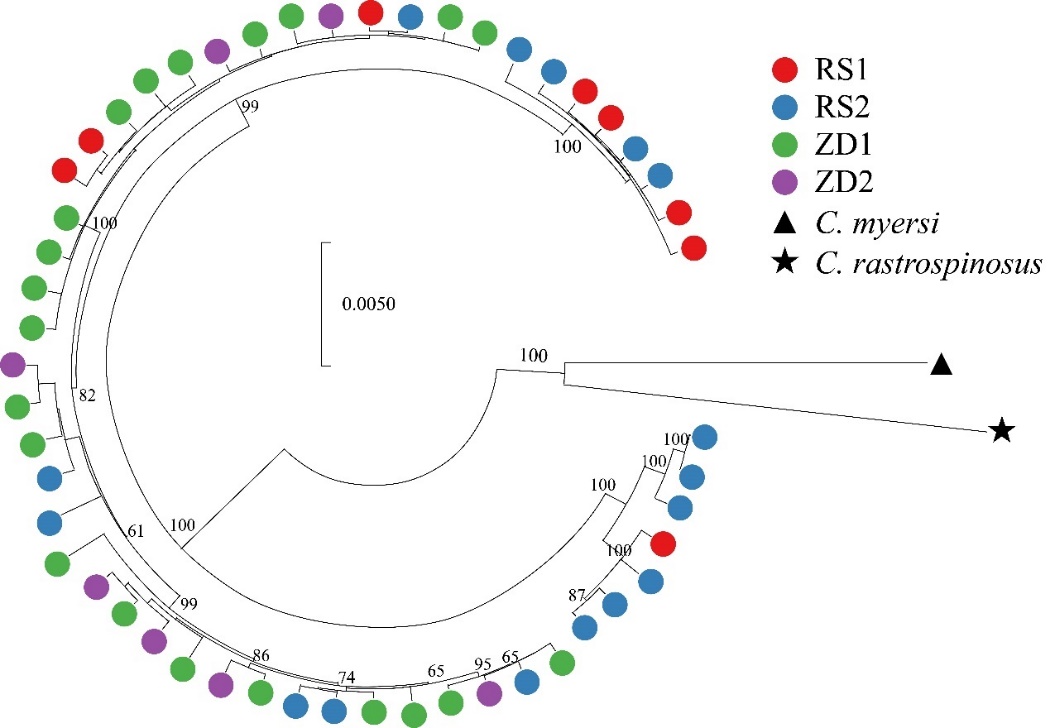


**Fig. S4.** Maxium likelihood trees reconstructed from extracted mitochondrial reads of 52 *C. hamastus* individuals together with *C. myersi* and *C. rastrospinosus*. The mitochondrial reads were extracted from the WGS data according to the alignments of the reads against the *C. hamatus* mitochondrial genome (Acession NC_029737.1). The SNPs identified with these mito-reads were used to construct a maximum likelihood phylogenetic tree (RAxML，v.8.2.12，-N 1000).The number on each branch shows the value of bootstrap. *C. myersi* (Accession NC_010689.1) and *C. rastrospinesus* (Accession NC_039543.1) were downloaded from the Genbank (Table S20).


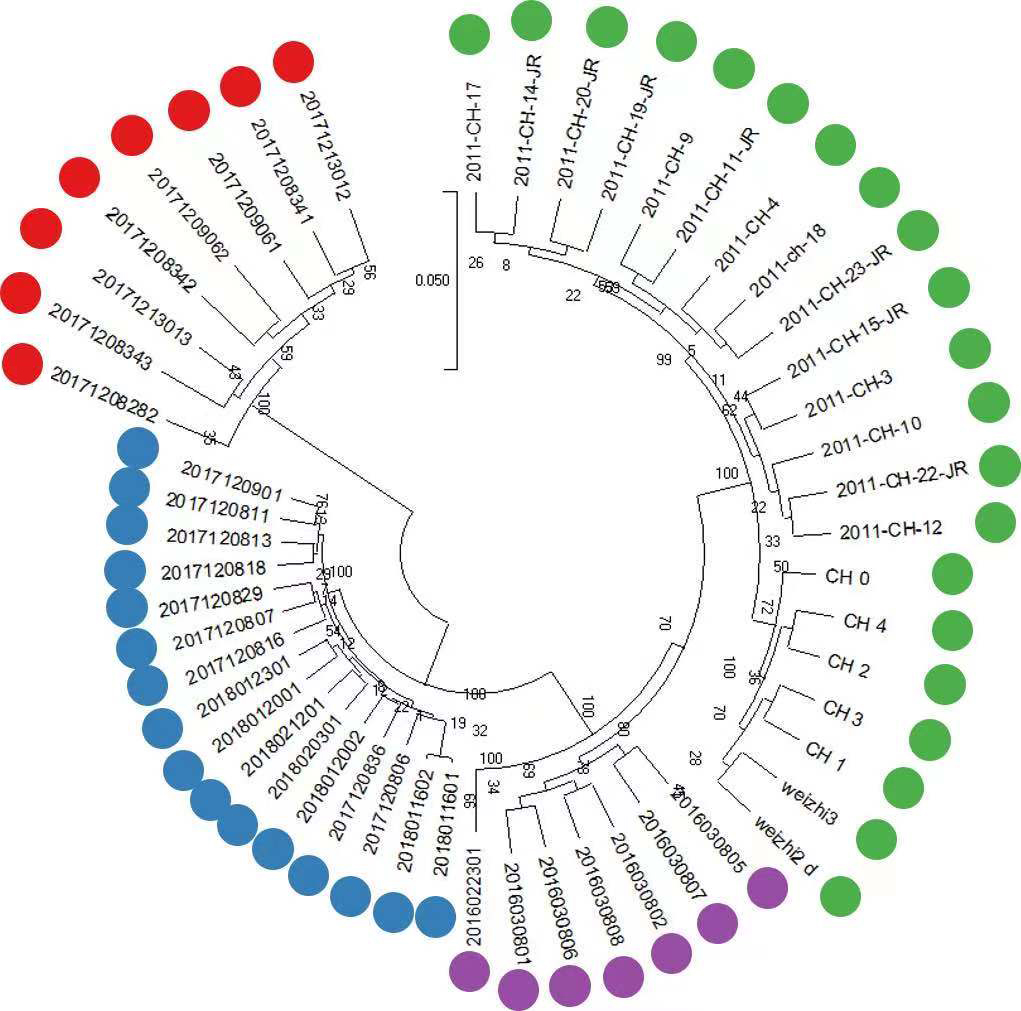


**Fig. S5.** The phylogeny of the re-sequenced 52 individuals with individual names shown supplementary to Fig.1C. The RS1, RS2, ZD1 and ZD2 populations are respectively presented with the filled circles in red, blue, purple and green. The numbers in black correspond to values of bootstrap support.


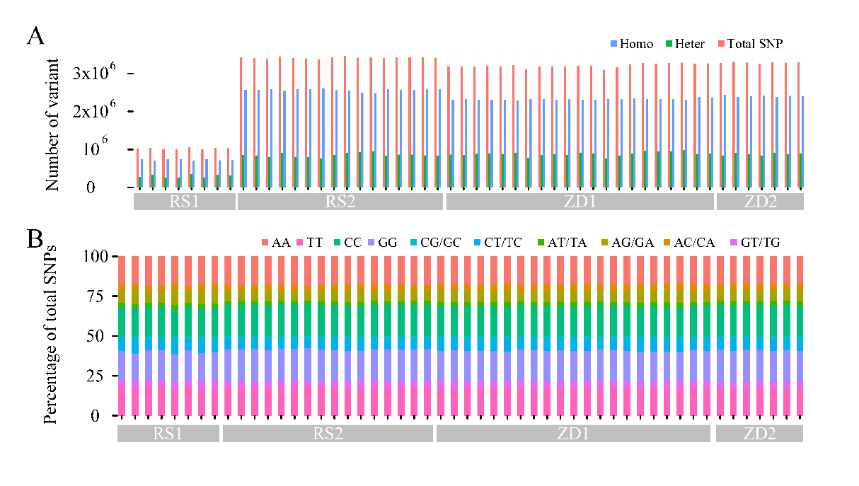


**Fig. S6.** Characteristics of SNPs identified in the *C. hamatus* populations. (A) Variant number for the 4 populations of 52 individuals. (B) Mutation spectrum for the 4 populations. The reference genome used is in this analysis was the high-quality *C. hamatus* reference genome assembled from the combination of third and second generation sequencing of the female individual appeared to be more closely related to RS1. This genome is used for all other analyses.


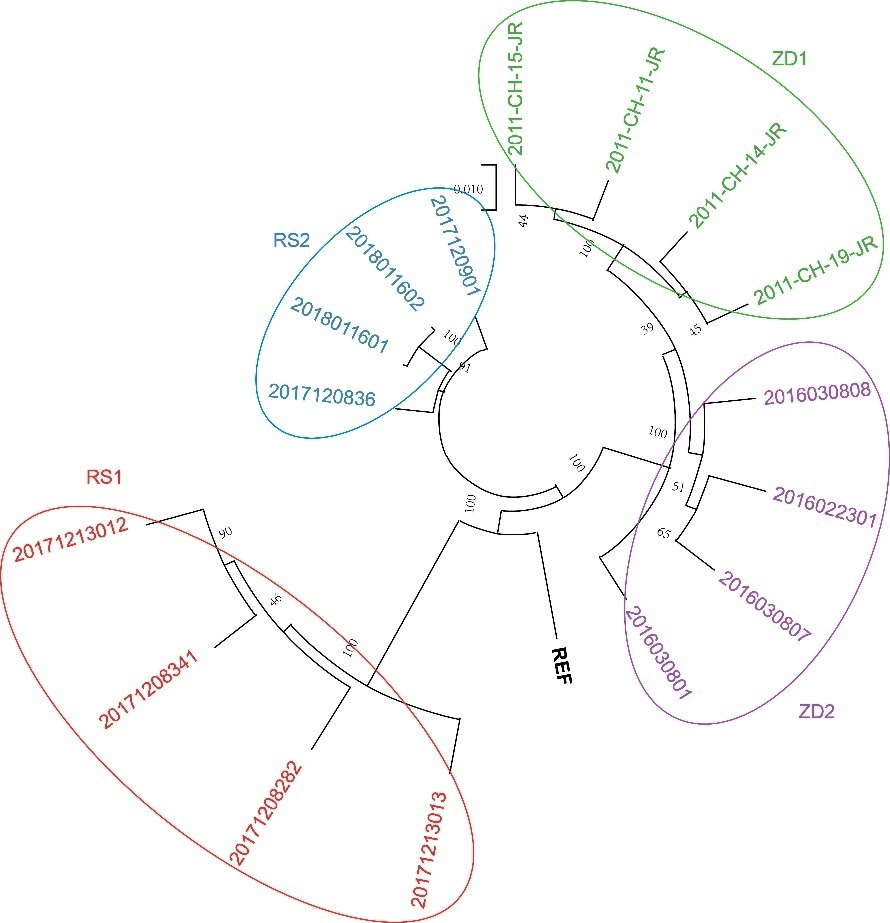


**Fig. S7.** Phylogeny tree depicts the closer relationship between RS1 and the individual of the reference genome (REF). Four individuals in each population were randomly chosen to reconstruct the phylogenetic tree with reference genome based on their SNPs using RAxML under the same parameters as Fig. 1C and Fig. S5.

**
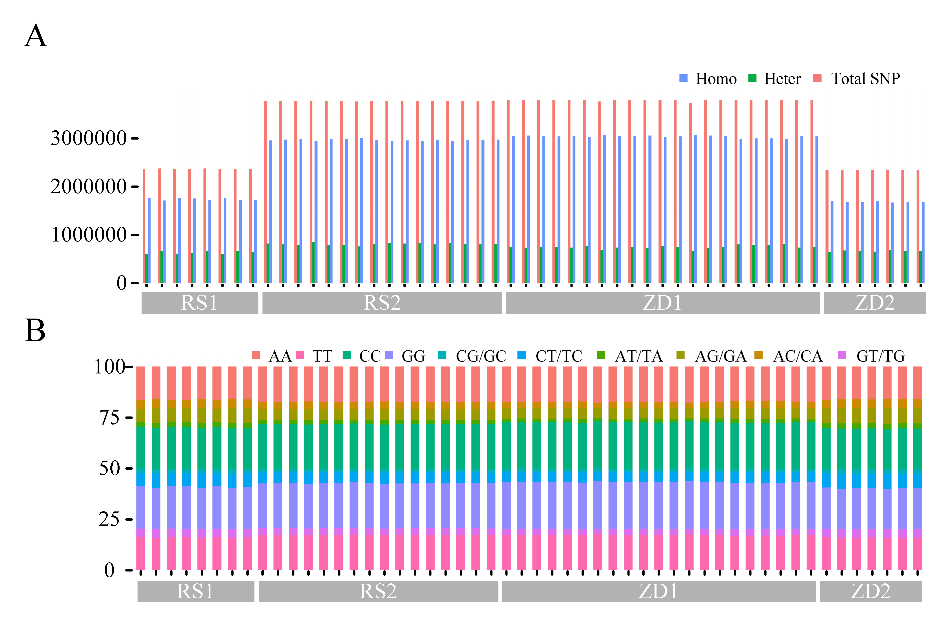
**

**Fig. S8.** Results of a parallel SNP call from the 52 individuals against an *ad-hoc* draft genome assembled from only the WGS sequencing reads from an ZD2 individual. (A) Variant number for the 4 populations of 52 individuals. (B) Mutation spectrum for the 4 populations.


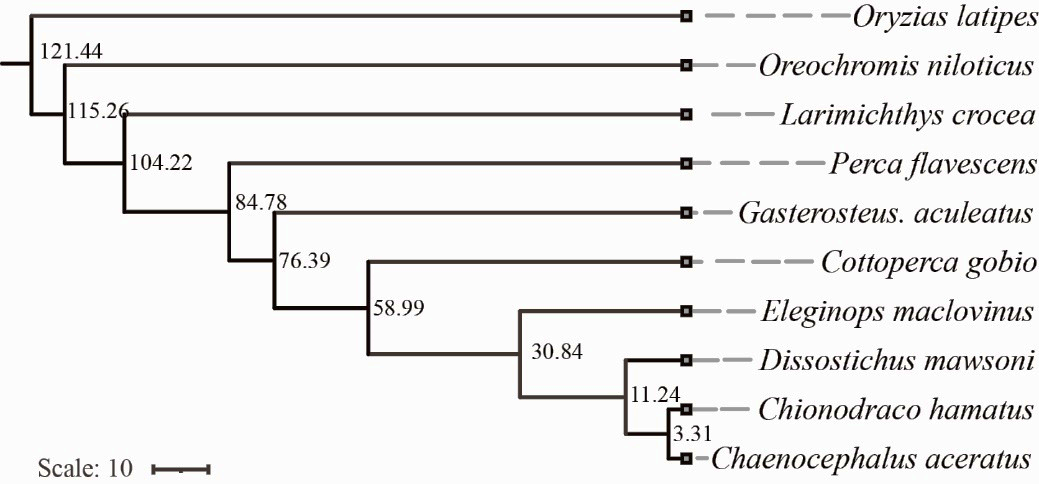


**Fig. S9** Reconstructed phylogenetic tree of ten fishes based on the maximum likelyhood method. The numbers shown on the nodes are estimated divergence time between species in millions of years (mya). The divergence time of 3.31 my between *C. hamatus* and *C. aceratus* were used to calculate the neutral mutation rate (μ) of *C. hamatus*.


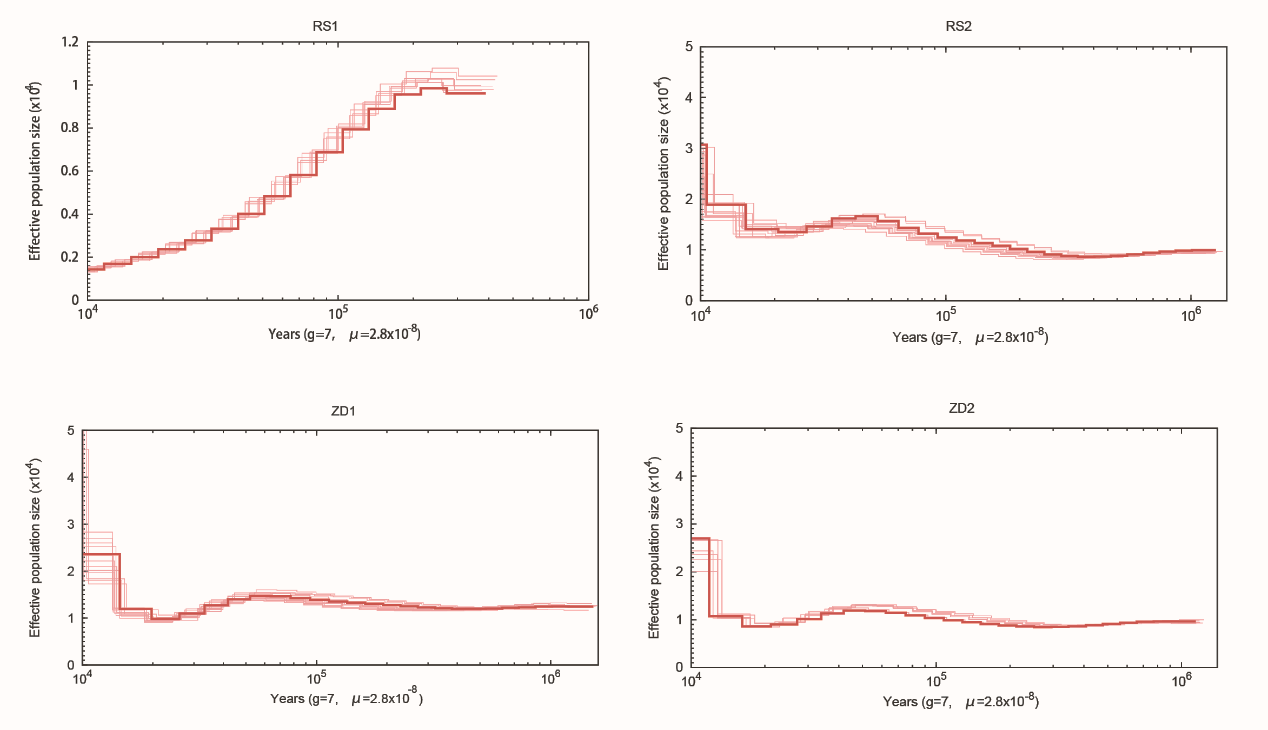


**Fig. S10.** The demographic histories of *C. hamatus* populations, RS1, RS2, ZD1, and ZD2, parallelly estimated by Pairwise sequentially Markovian coalescent (PSMC). The prameters used in the prediction were：-p: 25*2+4+6, -g: 7 and -u: 2.8487e-8.


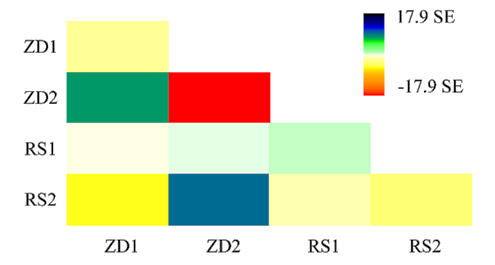


**Fig. S11.** Corresponding scaled residuals of Fig. 2F from the fit of the Treemix model to the data.


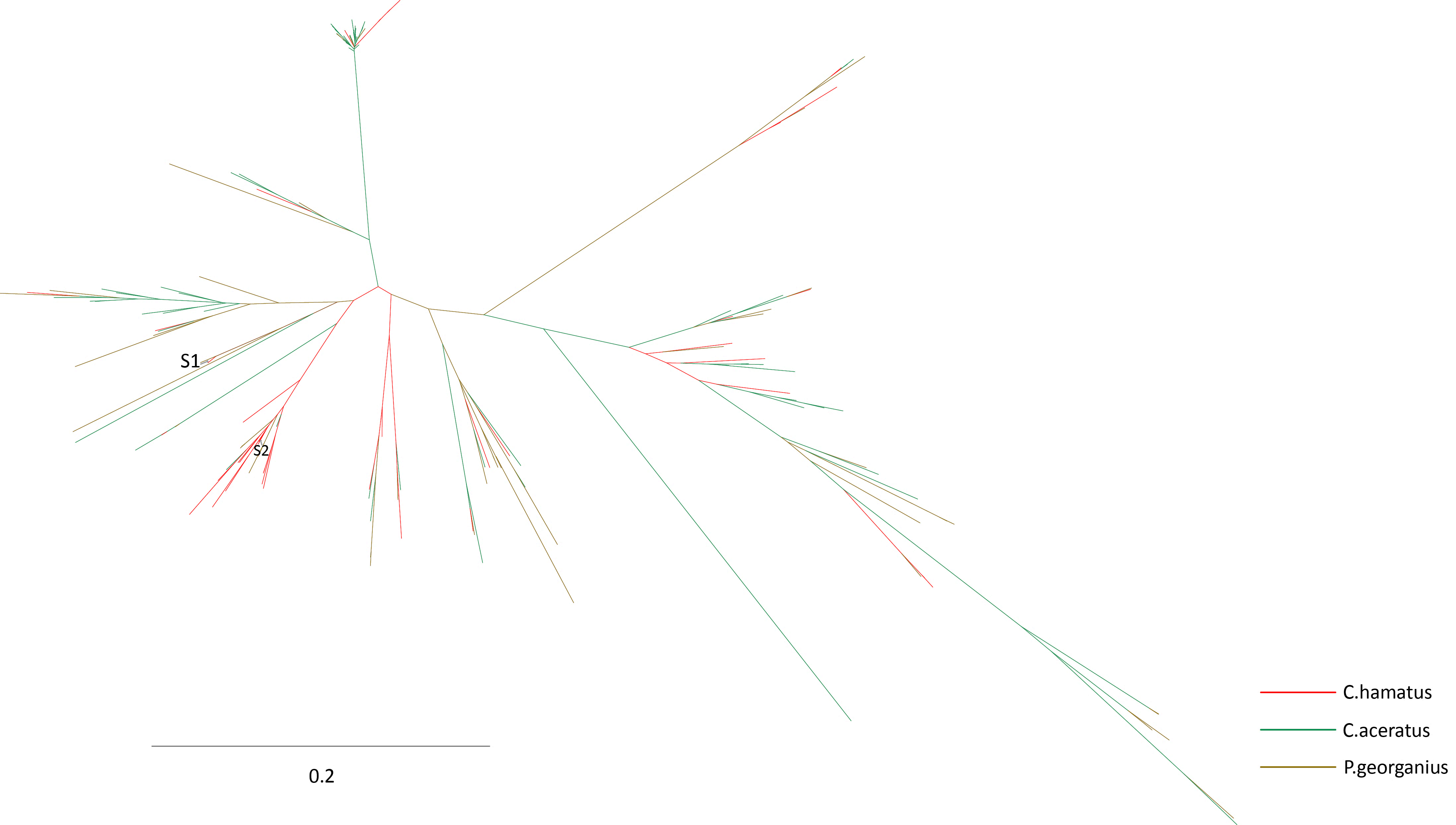


**Fig. S12.** The phylogenetic tree of the trim-35 and trim-39-like homologous genes from three icefish genomes indicating gene family dynamics between the species. S1 (Acc#OM337937) and S2 (Acc# OM337938) represent the homologous genes used for the antiviral activity studies shown in Fig. 3D.


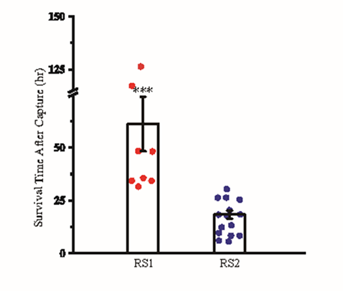


**Fig. S13.** Differential survival time after capture by RS1 and RS2. All fishes were kept in a 2x2x1 m^3^ well-oxygenated tank filled with seawater from the fishing site and maintained at a temperature close to that of the sea. The time lapse from the capture to loss of body equilibrium for the individual fishes was recorded. (one-way ANOVA, *** P < 0.001).

**
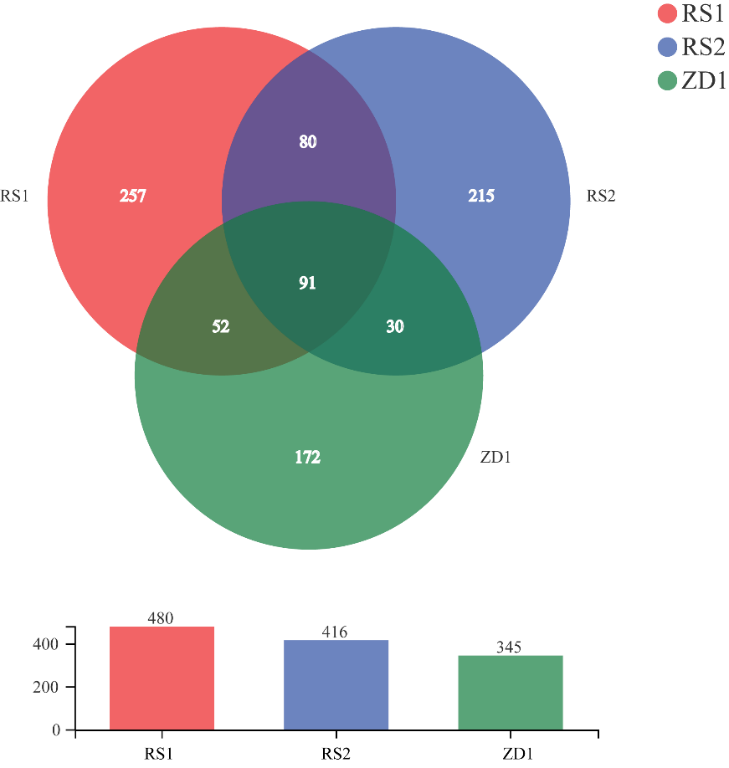
**

**Fig. S14.** The Venn diagram showing the total number of bacterial species identified in three populations.

**
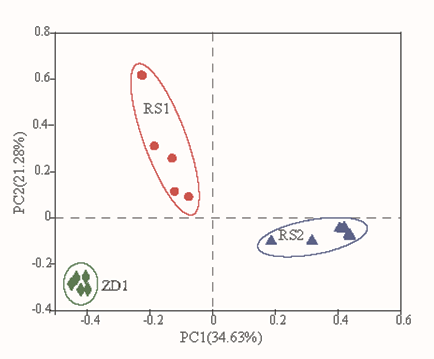
**

**Fig. S15.** Principal Component Analysis showing a population-based clustering pattern of the gut microbiota in the three populations.


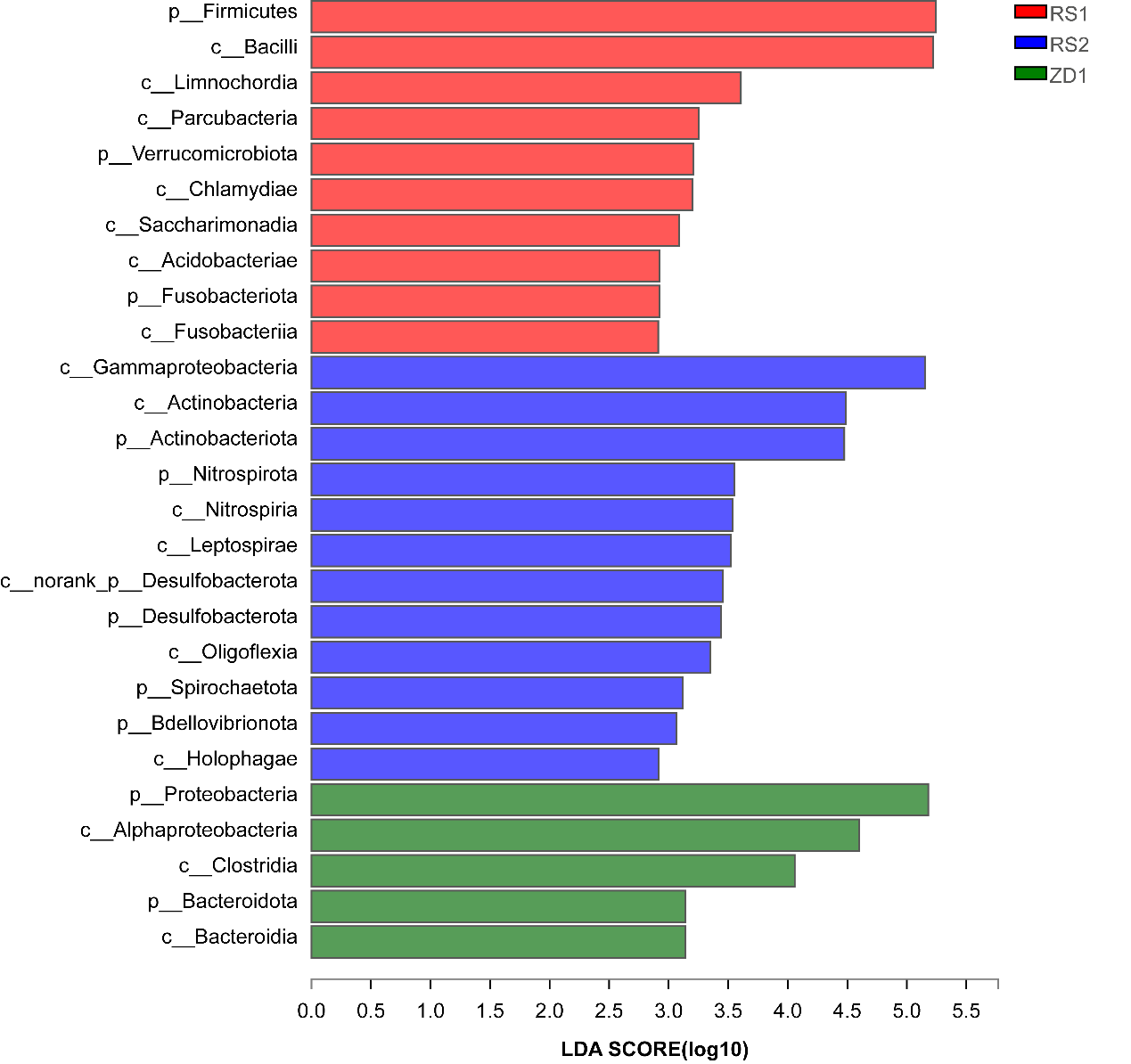


**Fig. S16.** Differentially abundant gut microbiota species specifically associated with RS1, RS2 and ZD1 as determined by LEfSe. Bacterial community at feature-level based on adjusted *P*-value cutoff = 0.05 with LDA score > 2.


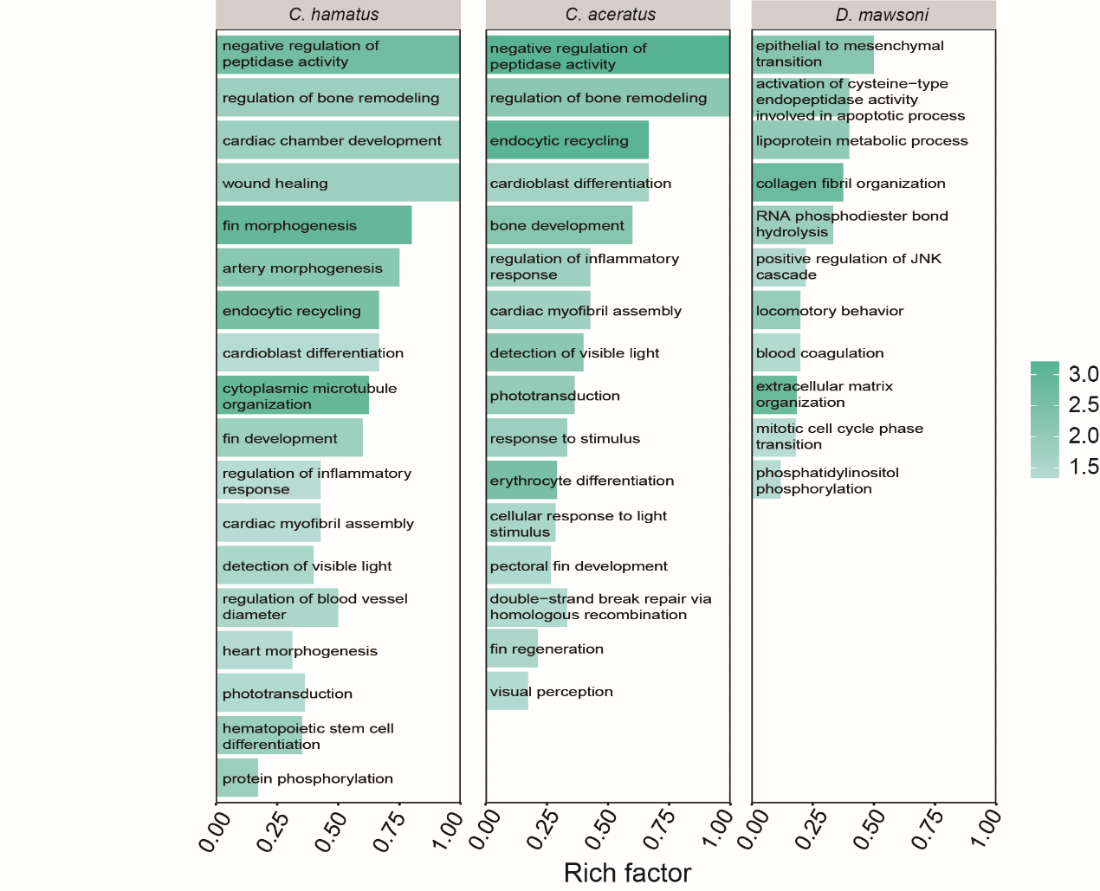


**Fig. S17.** The enriched GO list of the positive selection genes identified in *C. hamatus*, *C. aceratus* and *D. mawsoni,* supplementary to Fig. 4G.


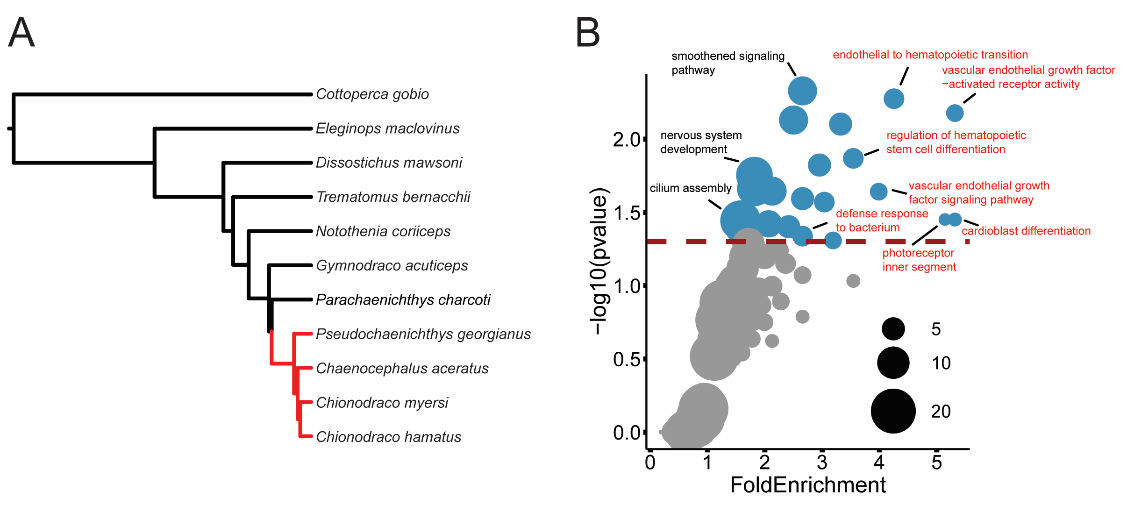


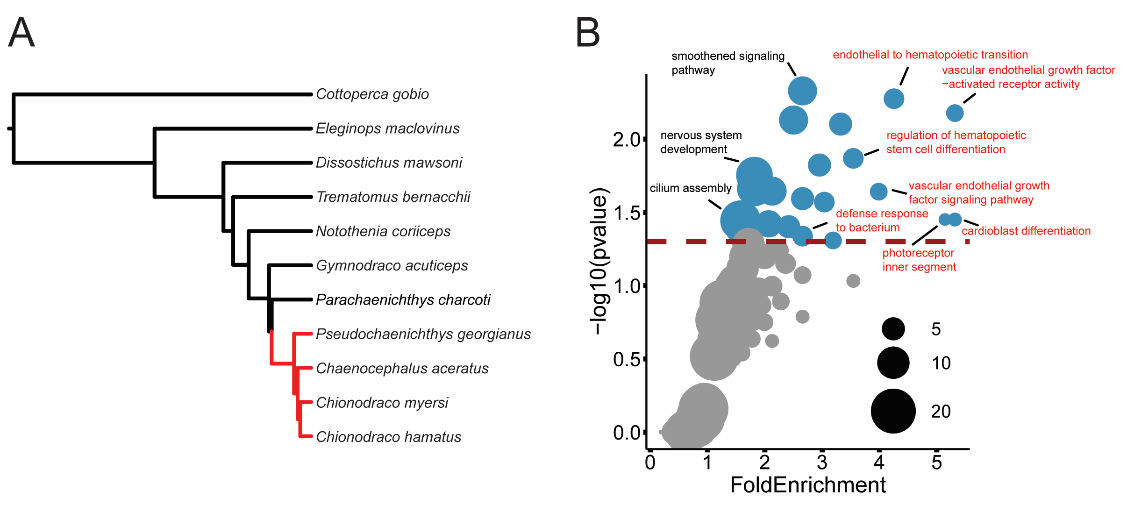


**Fig. S18.** Phylogenetic analysis and gene evolution of notothenioid fish. **(A)** The reconstructed phylogenetic relationships of eleven nototheoid fishes, *Cottoperca gobio*, *Eleginops maclovinus*, *Dissostichus mawsoni*, *Trematomus bernacchii*, *Notothenia coriiceps*, *Gymnodraco aculeatus*, *Parachaenichthys charcoti*, *Pseudochaenichthys georgianu*s, *Chaenocephalus aceratus*, *Chionodraco myersi* and *Chionodraco hamatus* used for PAML analysis. The tree is constructed based on a supergene constructed from 4000 orthologous genes of the species using RAxML. **( B)** A total of 711 genes (adjusted *P* < 0.05) with accelerated evolution was identified. Gene Ontology enrichment test on the accelerated evolution genes identified from the icefish lineage are shown, with the terms involved with cardiovascular development, visual perception and immune defense shown in red. The red dashed line indicates a *P* value = 0.05.


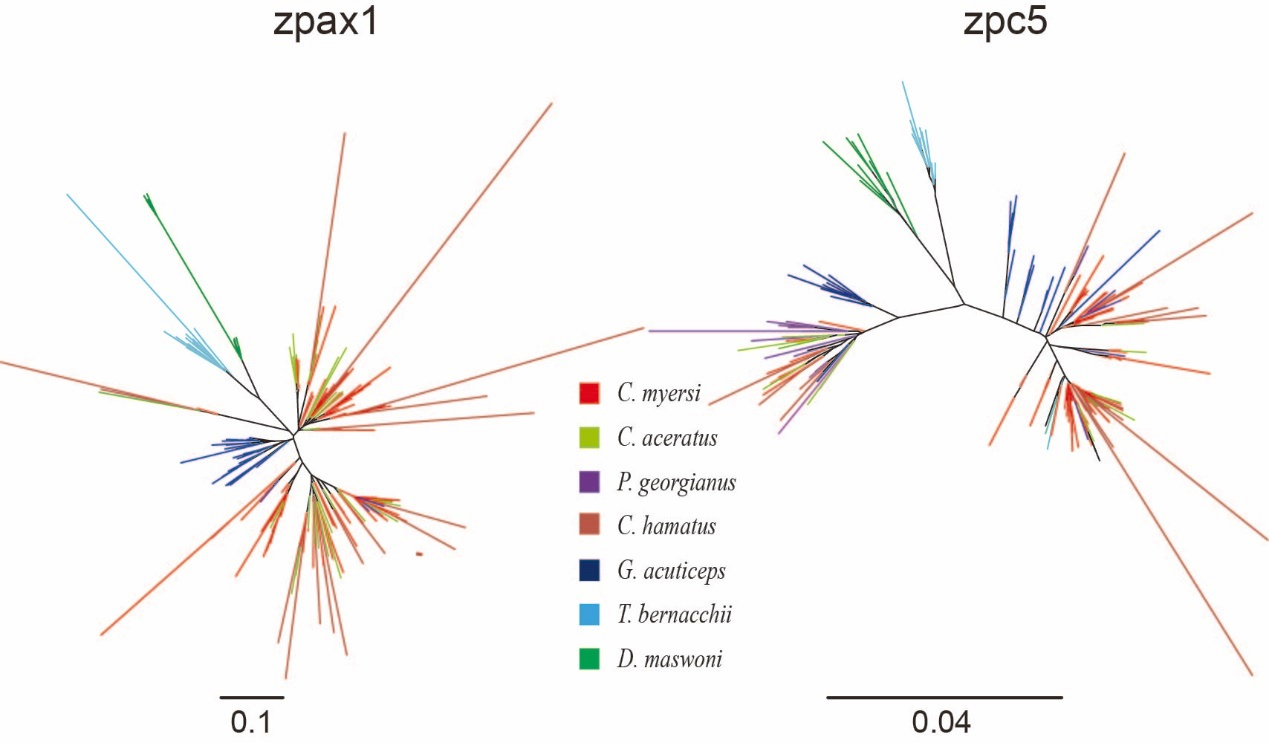


**Fig. S19.** Phylogenetic trees of the ZPAX1 and ZPC5 genes contained in the seven Antarctic species, indicating the more frequent expansions of the two gene families in the white-blooded icefish lineage, and the relative species-specific distribution and less duplication of the ZP genes in the red-blooded notothenioids.


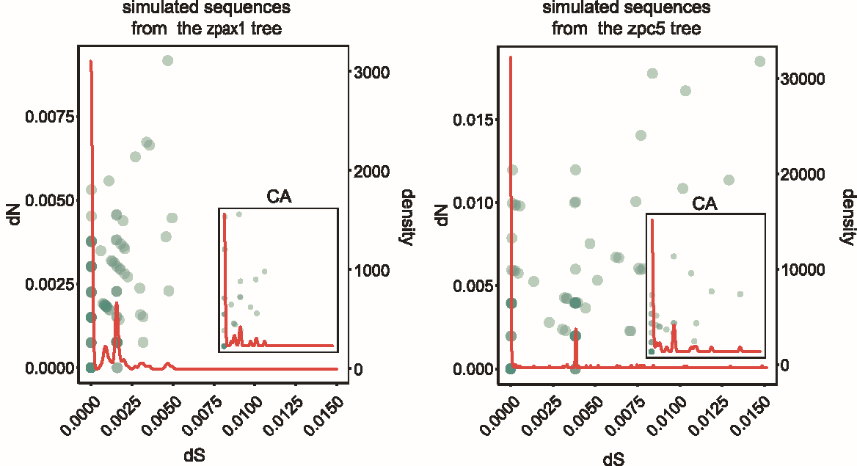


**Fig. S20.** The distribution of *d*S values of the simulated sequences from the ZPAX1 and ZPC5 phylogenetic trees. To evaluate the performance of free-ratio model in ZP gene family, the multiple sequence alignment (MSA) were simulated by Alisim that is a fast and versatile phylogenetic sequence simulator and an extension of the IQ-TREE. The periodic appearance of the *dS* peaks seen in the ZPAX1 and ZPC5 gene families of the Antarctic notothenioids shown in Fig. 4D-E disappeared in the simulated sequences derived from the same phylogenetic trees, suggested the periodic expansion of these gene families are unique features associated with the ZPAX1 and ZPC5 sequences. The regular dS distribution intervals disappeared in the simulated sequences derived from the trees of the *C. hamatus* ZPAX1 and ZPC5 gene sets (the inset image in each figure).


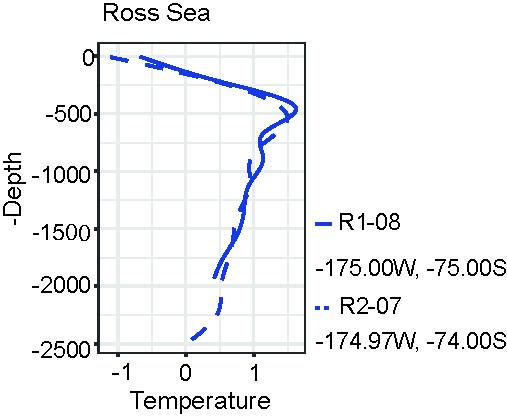


**Fig. S21.** The temperature curve along the Ocean depth, indicated the presence of nonfreezing temperatures at greater depths. The data is collected by the 34th Antarctic Exploration Team of China on January 26-28, 2018 at locations about 12 latitudes of distance to the R1-06 shown in Fig. 1D.

**Table S1** Statistics of sequencing data.

| **Types** | | **Method** | **Library Size (bp)** | **Clean data (Gb)** | **Length (bp)** | **Coverage (×)** |
| --- | --- | --- | --- | --- | --- | --- |
| Genomes | Genome | Illumina | 300-350 | 208.81 | 120 | 181.57 |
|  | Genome | PacBio | \ | 100.28 | \ | 87.20 |
|  | population | Illumina | 300-350 | 783.72 | 150 | 13.11 (for each sample) |
| Transcriptomes | | Illumina | 300-350 | 61.27 | 150 | 53.27 |

**Table S2** BUSCO Outputs for the *C. hamatus* genome.

|  | **CVG Gene Set** | **%** |
| --- | --- | --- |
| Complete BUSCOs (C) | 3,346 | 91.9 |
| Complete and single-copy BUSCOs (S) | 3,283 | 90.2 |
| Complete and duplicated BUSCOs (D) | 63 | 1.7 |
| Fragmented BUSCOs (F) | 76 | 2.1 |
| Missing BUSCOs (M) | 218 | 6.0 |
| Total BUSCO group searched | 3,640 | 100 |

Notes: BUSCO version is 5.0.0. The lineage dataset is: actinoperygii_odb10 (Creation date: 2020-08-05, number of species: 3640, number of BUSCOs: 26)

**Table S3** Annotated repetitive sequences.

| Transposable element |  | Repeats | Number | Length occupied (bp) | Percentages of sequences (%) |
| --- | --- | --- | --- | --- | --- |
|  | LTR retrotransposon | LTR/Copia | 8,893 | 4,656,257 | 0.40 |
|  |  | LTR/DIRS | 30,054 | 24,227,621 | 2.10 |
|  |  | LTR/ERV1 | 30,857 | 12,865,159 | 1.12 |
|  |  | LTR/ERVK | 3,719 | 1,182,016 | 0.10 |
|  |  | LTR/Gypsy | 26,647 | 16,247,826 | 1.41 |
|  |  | LTR/Gypsy-Cigr | 826 | 600,839 | 0.05 |
|  |  | LTR/Ngaro | 3,335 | 1,966,991 | 0.17 |
|  |  | LTR/Pao | 4,575 | 2,705,704 | 0.23 |
|  |  | Total | 108,906 | 64,452,413 | 5.60 |
|  | Non-LTR retrotransposon LINE | LINE/CR1 | 6,916 | 1,304,922 | 0.11 |
|  |  | LINE/I | 7,729 | 1,996,261 | 0.17 |
|  |  | LINE/I-Jockey | 2,211 | 467,947 | 0.04 |
|  |  | LINE/I-Nimb | 3,453 | 1,156,994 | 0.10 |
|  |  | LINE/Jockey | 468 | 238,420 | 0.02 |
|  |  | LINE/L1 | 5,285 | 2,421,407 | 0.21 |
|  |  | LINE/L1-Tx1 | 2,895 | 2,130,489 | 0.19 |
|  |  | LINE/L2 | 135,032 | 65,611,297 | 5.70 |
|  |  | LINE/Penelope | 163 | 10,333 | 0.00 |
|  |  | LINE/Proto2 | 1,184 | 438,878 | 0.04 |
|  |  | LINE/R2-Hero | 878 | 138,039 | 0.01 |
|  |  | LINE/R2-NeSL | 159 | 96,079 | 0.01 |
|  |  | LINE/Rex-Babar | 53,780 | 23,147,528 | 2.01 |
|  |  | LINE/RTE-BovB | 10,162 | 7,110,573 | 0.62 |
|  |  | LINE/RTE-X | 1,699 | 545,634 | 0.05 |
|  |  | Total | 232,014 | 106,814,801 | 9.28 |
|  | Non-LTR retrotransposon SINE | SINE | 6,487 | 1,005,150 | 0.09 |
|  |  | SINE? | 2,340 | 792,317 | 0.07 |
|  |  | SINE/ID | 0 | 0 | 0.00 |
|  |  | SINE/MIR | 17,517 | 2,769,890 | 0.24 |
|  |  | SINE/tRNA-Core-L2 | 2,864 | 363,766 | 0.03 |
|  |  | SINE/tRNA-L1 | 588 | 61,083 | 0.01 |
|  |  | Total | 29,796 | 4,992,206 | 0.43 |
|  | DNA transposon | DNA | 51,614 | 1,3733,349 | 1.19 |
|  |  | DNA/Academ | 506 | 294,985 | 0.03 |
|  |  | DNA/Academ-1 | 2,300 | 495,139 | 0.04 |
|  |  | DNA/CMC-EnSpm | 13,101 | 4,489,244 | 0.39 |
|  |  | DNA/Crypton | 4,216 | 1,153,581 | 0.10 |
|  |  | DNA/Crypton-A | 2,282 | 375,589 | 0.03 |
|  |  | DNA/Crypton-V | 7,406 | 1,171,135 | 0.10 |
|  |  | DNA/Ginger | 267 | 137,857 | 0.01 |
|  |  | DNA/hAT | 4,617 | 1,621,102 | 0.14 |
|  |  | DNA/hAT-Ac | 173,837 | 56,464,504 | 4.90 |
|  |  | DNA/hAT-Blackjack | 10,625 | 2,924,834 | 0.25 |
|  |  | DNA/hAT-Charlie | 74,350 | 23,562,711 | 2.05 |
|  |  | DNA/hAT-hAT5 | 10,134 | 2,824,703 | 0.25 |
|  |  | DNA/hAT-hATx | 777 | 123,225 | 0.01 |
|  |  | DNA/hAT-hobo | 2,418 | 730,707 | 0.06 |
|  |  | DNA/hAT-Pegasus | 0 | 0 | 0.00 |
|  |  | DNA/hAT-Tip100 | 53,928 | 15,918,145 | 1.38 |
|  |  | DNA/hAT-Tol2 | 0 | 0 | 0.00 |
|  |  | DNA/IS3EU | 9,924 | 2,998,049 | 0.26 |
|  |  | DNA/Kolobok-T2 | 40,118 | 11,224,185 | 0.97 |
|  |  | DNA/Maverick | 3,780 | 1,522,309 | 0.13 |
|  |  | DNA/Merlin | 1,977 | 418,668 | 0.04 |
|  |  | DNA/MULE-MuDR | 0 | 0 | 0.00 |
|  |  | DNA/Novosib | 0 | 0 | 0.00 |
|  |  | DNA/P | 25,909 | 13,310,086 | 1.16 |
|  |  | DNA/PIF-Harbinger | 47,653 | 18,140,922 | 1.58 |
|  |  | DNA/PIF-ISL2EU | 4,031 | 2,074,821 | 0.18 |
|  |  | DNA/PiggyBac | 13,406 | 4,349,161 | 0.38 |
|  |  | DNA/Sola-1 | 0 | 0 | 0.00 |
|  |  | DNA/Sola-2 | 1,256 | 558,879 | 0.05 |
|  |  | DNA/TcMar-Fot1 | 1,011 | 343,264 | 0.03 |
|  |  | DNA/TcMar-ISRm11 | 2,420 | 754,979 | 0.07 |
|  |  | DNA/TcMar-Stowaway | 425 | 203,833 | 0.02 |
|  |  | DNA/TcMar-Tc1 | 18,688 | 8,257,983 | 0.72 |
|  |  | DNA/TcMar-Tc2 | 2,357 | 601,071 | 0.05 |
|  |  | DNA/Zisupton | 20,650 | 12,282,321 | 1.07 |
|  |  | RC/Helitron | 12,035 | 5,212,326 | 0.45 |
|  |  | Total | 618,018 | 208,273,667 | 18.09 |
| Unclassified | | | 443,473 | 141,948,494 | 12.33% |
| Total Interspersed | | | 1,432,207 | 526,481,581 | 45.72% |
| Satellite | | | 29,446 | 5,688,101 | 0.49% |
| Simple_repeat | | | 525,505 | 39,735,428 | 3.45% |
| Low_complexity | | | 65,268 | 4,196,571 | 0.36% |
| Total | | | 2,052,426 | 576,101,681 | 50.03% |

**Table S4** Statistics of the assemblies for each pseudo-chromosome.

| **pseudo-chromosome ID** | **Contig No.** | **Length (bp)** |
| --- | --- | --- |
| LG1 | 25 | 45,184,151 |
| LG2 | 11 | 13,750,805 |
| LG3 | 21 | 45,213,784 |
| LG4 | 13 | 43,431,246 |
| LG5 | 18 | 40,397,750 |
| LG6 | 16 | 40,246,285 |
| LG7 | 32 | 43,233,497 |
| LG8 | 14 | 32,765,423 |
| LG9 | 23 | 44,842,316 |
| LG10 | 19 | 34,945,683 |
| LG11 | 11 | 26,796,043 |
| LG12 | 10 | 24,466,198 |
| LG13 | 21 | 40,183,158 |
| LG14 | 15 | 32,625,023 |
| LG15 | 23 | 34,966,442 |
| LG16 | 22 | 41,719,997 |
| LG17 | 25 | 34,536,362 |
| LG18 | 12 | 18,555,584 |
| LG19 | 12 | 24,432,279 |
| LG20 | 12 | 24,122,679 |
| LG21 | 28 | 33,917,015 |
| LG22 | 21 | 32,688,869 |
| LG23 | 11 | 22,276,069 |
| LG24 | 17 | 30,748,931 |

**Table S5.** Functional classification of the protein-coding genes according to different databases.

|  | | **Number** | **Percent (%)** |
| --- | --- | --- | --- |
| Total | | 30,266 | 100 |
| Annotated | Interproscan | 17,935 | 59.26 |
|  | KOG | 19,662 | 64.96 |
|  | NR | 25,273 | 83.50 |
|  | Swissport | 18,505 | 61.14 |
|  | TrEMBL | 19,710 | 65.12 |
| Unannotated | | 26,255 | 86.75 |

**Table S6.** Statistics of protein-coding genes predicted by various methods.

| Gene set | | | Number | Average transcript length (bp) | Average CDS length (bp) | Average exons per gene | Average exon length (bp) | Average intron length (bp) |
| --- | --- | --- | --- | --- | --- | --- | --- | --- |
| *De novo* | SNAP | | 114,138 | 11,775 | 633 | 4.30 | 147 | 3,367 |
|  | AUGUSTUS | | 57,278 | 6,862 | 913 | 4.73 | 193 | 1,595 |
| Homolog | Exo-nerate | *D.mawsoni* | 46,386 | 12,701 | 196 | 1.51 | 170 | 2,685 |
|  |  | *C.aceratus* | 163,911 | 8,483 | 521 | 3.06 | 170 | 3,857 |
|  |  | *D.rerio* | 33,246 | 16,662 | 1,056 | 6.25 | 169 | 2,965 |
| Transcriptome | Trinity | PASA | 109,262 | 14,756 | 800 | 8.19 | 307 | 1,702 |
|  | Cufflinks |  |  |  |  |  |  |  |
| EVidenceModeler | | | 30,266 | 12,430 | 1,371 | 7.20 | 190 | 1,783 |

**Table S7.** Summary of non-coding RNA genes in *C. hamatus* genome.

| **ncRNA** | **Number in of loci** | **Average length (bp)** | **Total length (bp)** | **% of assemblies** |
| --- | --- | --- | --- | --- |
| tRNA | 4,926 | 73.61 | 362,596 | 0.0315 |
| rRNA | 705 | 960.45 | 677,119 | 0.0588 |
| miRNA | 2,944 | 130.30 | 383,598 | 0.0333 |
| snRNA | 917 | 147.21 | 134,990 | 0.0117 |
| snoRNA |  |  |  |  |
| CD-box RNA | 501 | 127.68 | 63,966 | 0.0056 |
| HACA-box RNA | 449 | 217.60 | 97,704 | 0.0085 |

**Table S8**. Collected *C. hamatus* samples and sequencing statistics.

| **Popu-lation ID** | **Sample ID** | **No. of reads** | **Total data (bp)** | **Mapping rate of reads (%)** | **Seq.**  **Depth (x)** | **GPS coordinates** | **Fishing Depth (m)** |
| --- | --- | --- | --- | --- | --- | --- | --- |
| **RS1** | 20171208282 | 112,931,784 | 14,094,272,700 | 98.03 | 12.2 | 74°55′S, 163°46′E | 130 |
|  | 20171208341 | 128,850,532 | 16,154,795,700 | 98.28 | 14.0 | 74°55′S, 163°46′E | 130 |
|  | 20171208342 | 101,858,119 | 12,979,428,900 | 98.44 | 11.3 | 74°55′S, 163°46′E | 130 |
|  | 20171208343 | 102,662,800 | 13,031,652,600 | 98.49 | 11.3 | 74°55′S, 163°46′E | 130 |
|  | 20171209061 | 146,364,709 | 18,616,109,400 | 98.45 | 16.2 | 74°55′S, 163°46′E | 130 |
|  | 20171209062 | 103,193,757 | 13,087,630,500 | 98.48 | 11.4 | 74°55′S, 163°46′E | 130 |
|  | 20171213012 | 119,208,306 | 15,126,395,400 | 98.55 | 13.1 | 74°55′S, 163°46′E | 130 |
|  | 20171213013 | 116,991,581 | 14,838,282,000 | 98.47 | 12.9 | 74°55′S, 163°46′E | 130 |
| **RS2** | 2017120806 | 105,169,948 | 14,716,050,600 | 93.00 | 12.8 | 74°55′S, 163°46′E | 130 |
|  | 2017120807 | 104,289,756 | 14,567,349,600 | 94.03 | 12.7 | 74°55′S, 163°46′E | 130 |
|  | 2017120811 | 104,716,084 | 14,588,844,600 | 92.45 | 12.7 | 74°55′S, 163°46′E | 130 |
|  | 2017120813 | 118,432,161 | 16,558,308,900 | 93.96 | 14.4 | 74°55′S, 163°46′E | 130 |
|  | 2017120816 | 106,384,956 | 14,818,705,500 | 92.87 | 12.9 | 74°55′S, 163°46′E | 130 |
|  | 2017120818 | 106,086,564 | 14,780,012,700 | 92.81 | 12.8 | 74°55′S, 163°46′E | 130 |
|  | 2017120829 | 104,188,800 | 14,498,117,700 | 92.34 | 12.6 | 74°55′S, 163°46′E | 130 |
|  | 2017120836 | 112,604,606 | 15,712,758,000 | 92.20 | 13.6 | 74°55′S, 163°46′E | 130 |
|  | 2017120901 | 122,082,442 | 17,091,118,200 | 94.03 | 14.8 | 74°55′S, 163°46′E | 130 |
|  | 2018011601 | 103,062,250 | 14,444,784,300 | 94.33 | 12.5 | 74°56′S, 163°46′E | 120 |
|  | 2018011602 | 108,125,579 | 15,099,223,800 | 93.74 | 13.1 | 74°56′S, 163°46′E | 120 |
|  | 2018012001 | 107,654,111 | 15,006,561,000 | 93.14 | 13.0 | 74°55′S, 163°47′E | 130 |
|  | 2018012002 | 108,312,406 | 15,145,290,300 | 94.12 | 13.2 | 74°55′S, 163°47′E | 130 |
|  | 2018012301 | 107,633,857 | 15,048,761,100 | 93.99 | 13.1 | 74°55′S, 163°47′E | 130 |
|  | 2018020301 | 108,863,455 | 15,200,337,000 | 93.75 | 13.2 | 74°56′S, 163°46′E | 130 |
|  | 2018021201 | 107,984,690 | 15,069,652,800 | 94.26 | 13.1 | 74°55′S, 163°47′E | 130 |
| **ZD1** | 2011-CH-10 | 126,902,207 | 16,614,800,700 | 94.03 | 14.4 | 68°34'42"S, 77°57'60"E | 30 |
|  | 2011-CH-11-JR | 115,369,604 | 15,046,966,500 | 94.33 | 13.1 | 68°34'42"S, 77°57'60"E | 30 |
|  | 2011-CH-12 | 121,573,531 | 15,894,798,000 | 94.10 | 13.8 | 68°34'42"S, 77°57'60"E | 30 |
|  | 2011-CH-14-JR | 116,989,783 | 15,284,080,200 | 94.38 | 13.3 | 68°34'42"S, 77°57'60"E | 30 |
|  | 2011-CH-15-JR | 112,136,499 | 14,635,413,900 | 94.10 | 12.7 | 68°34'42"S, 77°57'60"E | 30 |
|  | 2011-CH-17 | 144,349,634 | 18,863,104,200 | 93.96 | 16.4 | 68°34'42"S, 77°57'60"E | 30 |
|  | 2011-CH-19-JR | 110,404,126 | 14,406,711,900 | 94.01 | 12.5 | 68°34'42"S, 77°57'60"E | 30 |
|  | 2011-CH-20-JR | 116,511,804 | 15,172,624,200 | 94.09 | 13.2 | 68°34'42"S, 77°57'60"E | 30 |
|  | 2011-CH-22-JR | 116,046,190 | 15,143,658,300 | 94.37 | 13.2 | 68°34'42"S, 77°57'60"E | 30 |
|  | 2011-CH-23-JR | 115,372,002 | 15,037,797,000 | 94.40 | 13.1 | 68°34'42"S, 77°57'60"E | 30 |
|  | 2011-CH-3 | 128,828,266 | 16,853,028,000 | 94.06 | 14.6 | 68°34'42"S, 77°57'60"E | 30 |
|  | 2011-CH-4 | 121,949,365 | 15,936,472,200 | 94.22 | 13.8 | 68°34'42"S, 77°57'60"E | 30 |
|  | 2011-CH-9 | 122,344,640 | 15,970,367,400 | 94.02 | 13.9 | 68°34'42"S, 77°57'60"E | 30 |
|  | 2011-ch-18 | 114,589,671 | 14,928,216,300 | 94.16 | 13.0 | 68°34'42"S, 77°57'60"E | 30 |
|  | CH_0 | 123,020,965 | 14,070,241,938 | 89.64 | 12.2 | 68°34'42"S, 77°57'60"E | 30 |
|  | CH_1 | 101,788,142 | 14,957,194,200 | 96.65 | 13.0 | 68°34'42"S, 77°57'60"E | 30 |
|  | CH_2 | 84,308,206 | 12,363,528,000 | 96.17 | 10.7 | 68°34'42"S, 77°57'60"E | 30 |
|  | CH_3 | 96,505,047 | 14,156,372,400 | 96.70 | 12.3 | 68°34'42"S, 77°57'60"E | 30 |
|  | CH_4 | 110,443,464 | 16,225,361,400 | 96.46 | 14.1 | 68°34'42"S, 77°57'60"E | 30 |
|  | weizhi2 | 96,447,072 | 14,246,428,200 | 96.72 | 12.4 | 68°34'42"S, 77°57'60"E | 30 |
|  | weizhi3 | 102,354,208 | 15,082,925,700 | 97.02 | 13.1 | 68°34'42"S, 77°57'60"E | 30 |
| **ZD2** | 2016022301 | 104,411,361 | 14,508,906,600 | 93.26 | 12.6 | 66°16′S, 110°28′E | 30 |
|  | 2016030801 | 107,443,467 | 14,981,478,000 | 94.00 | 13.0 | 68°33′40″S, 77°53′6″E | 20 |
|  | 2016030802 | 107,081,333 | 14,890,166,400 | 93.50 | 12.9 | 68°33′40″S, 77°53′6″E | 20 |
|  | 2016030805 | 102,405,031 | 14,196,483,900 | 93.54 | 12.3 | 68°33′40″S, 77°53′6″E | 20 |
|  | 2016030806 | 108,772,860 | 15,118,077,600 | 94.88 | 13.1 | 68°33′40″S, 77°53′6″E | 20 |
|  | 2016030807 | 104,917,606 | 14,578,302,600 | 93.96 | 12.7 | 68°33′40″S, 77°53′6″E | 20 |
|  | 2016030808 | 102,484,841 | 14,277,245,100 | 94.63 | 12.4 | 68°33′40″S, 77°53′6″E | 20 |

**Table S9**. SNP calling in 4 populations.

| **Category** | **RS1** | **RS2** | **ZD1** | **ZD2** | **total** |
| --- | --- | --- | --- | --- | --- |
| Sample Size | 8 | 16 | 21 | 7 | 52 |
| Number of total SNPs | 1,132,340 | 3,582,300 | 3,368,176 | 3,399,124 | **11,481,940** |
| Number of shared SNPs | 122,208 | | | |  |

**Table S10** Tracy-Widom (TW) statistics for the first four eigenvalues from PCA analysis.

| **Eigenvectors** | **Eigen-value** | **Tw-state** | ***P* value** |
| --- | --- | --- | --- |
| 1 | 3.454410 | 7.199 | 7.91e-08 |
| 2 | 2.006540 | 7.378 | 4.78e-08 |
| 3 | 1.299840 | -1.706 | 0.638498 |
| 4 | 1.260780 | -1.673 | 0.628244 |

**Table S11.** The RS1 specific gene list under selective sweep. (genes with * are discussed in the manuscript)

| Gene ID | annotation |
| --- | --- |
| CH_00047_00688122 | glypican-6-like isoform X1 [Monopterus albus] |
| CH_00065_05518329 | hypothetical protein D623_10024738 [Myotis brandtii] |
| CH_00065_13341480 | LOW QUALITY PROTEIN: zinc finger MYM-type protein 4-like [Seriola dumerili] |
| CH_00067_00501943 | *putative serine palmitoyltransferase 2-like [Scophthalmus maximus] |
| CH_00134_00665800 | Retrovirus-related Pol polyprotein from transposon 412 [Labeo rohita] |
| CH_00139_01446587 | LINE-1 type transposase domain-containing protein 1 [Anabarilius grahami] |
| CH_00184_01099544 | hypothetical protein EPR50_G00054980 [Perca flavescens] |
| CH_00191_00045314 | *PREDICTED: LOW QUALITY PROTEIN: nuclear factor 7, brain-like [Notothenia coriiceps] |
| CH_00349_00425245 | hypothetical protein EPR50_G00235320 [Perca flavescens] |
| CH_00665_00127650 | polyprotein [Chionodraco hamatus] |
| CH_00701_00048345 | *zinc finger protein RFP-like isoform X3 [Perca flavescens]; XP_028460617.1 zinc finger protein RFP-like isoform X3 [Perca flavescens]; XP_028460624.1 zinc finger protein RFP-like isoform X3 [Perca flavescens] |
| CH_00701_00116347 | *E3 ubiquitin-protein ligase TRIM21-like [Perca flavescens] |
| CH_00701_00274541 | *E3 ubiquitin-protein ligase TRIM39-like [Perca flavescens] |
| CH_00701_00337840 | *E3 ubiquitin-protein ligase TRIM21-like [Perca flavescens] |
| CH_00701_00390318 | *E3 ubiquitin-protein ligase TRIM21-like isoform X2 [Perca flavescens] |
| CH_00717_00231226 | protocadherin Fat 2 [Perca flavescens]; TDH07324.1 hypothetical protein EPR50_G00104790 [Perca flavescens] |
| CH_00745_00309884 | hypothetical protein cypCar_00033470 [Cyprinus carpio] |
| CH_00868_00107525 | uncharacterized protein LOC109989458 [Labrus bergylta] |
| CH_00926_00004021 | *PREDICTED: tripartite motif-containing protein 35-like [Notothenia coriiceps] |
| CH_01451_00009773 | PREDICTED: serine/threonine-protein phosphatase 2B catalytic subunit gamma isoform-like isoform X1 [Clupea harengus] |
| CH_01713_00007944 | zinc finger CCCH domain-containing protein 14 isoform X2 [Perca flavescens] |
| CH_01824_00016930 | *PREDICTED: ladderlectin-like [Notothenia coriiceps] |
| CH_01872_00027763 | Unknow |
| CH_02855_00010213 | *LOW QUALITY PROTEIN: NACHT, LRR and PYD domains-containing protein 4-like [Perca flavescens] |
| CH_04424_00029964 | *E3 ubiquitin-protein ligase TRIM21-like [Perca flavescens] |
| CH_04763_00000946 | polyprotein [Chionodraco hamatus] |

**Table S12.** The RS2 specific gene list under selective sweep. (genes with * are discussed in the manuscript)

| Gene ID | annotation |  |
| --- | --- | --- |
| CH_00008_03133903 | *tyrosine-protein kinase JAK1 [Seriola dumerili] | |
| CH_00008_04327601 | *MAP kinase-interacting serine threonine- kinase 2 [Labeo rohita] | |
| CH_00008_04704550 | *serine/threonine-protein kinase Nek7 isoform X1 [Astatotilapia calliptera] | |
| CH_00008_04775876 | hypothetical protein CCH79_00009394 [Gambusia affinis] | |
| CH_00008_04989723 | PREDICTED: protein FAM163A [Notothenia coriiceps] | |
| CH_00008_05003079 | rab GTPase-activating protein 1-like isoform X1 [Anabas testudineus]; XP_026200956.1 rab GTPase-activating protein 1-like isoform X1 [Anabas testudineus] | |
| CH_00016_01045234 | *PREDICTED: R-spondin-3-like [Stegastes partitus] | |
| CH_00016_01052259 | PREDICTED: HD domain-containing protein 2 [Notothenia coriiceps] | |
| CH_00021_01449267 | lipoyl synthase, mitochondrial [Labrus bergylta]; XP_020481449.1 lipoyl synthase, mitochondrial-like [Labrus bergylta] | |
| CH_00021_01462569 | PREDICTED: replication factor C subunit 1 isoform X1 [Hippocampus comes] | |
| CH_00021_01552596 | uncharacterized protein LOC114570119 isoform X1 [Perca flavescens]; TDH16173.1 hypothetical protein EPR50_G00016950 [Perca flavescens] | |
| CH_00021_01572973 | PREDICTED: protein NOXP20 [Notothenia coriiceps] | |
| CH_00021_01623346 | PREDICTED: TBC1 domain family member 1 [Notothenia coriiceps] | |
| CH_00021_01699753 | putative phosphoglucomutase-2 [Scophthalmus maximus] | |
| CH_00047_03211222 | sn1-specific diacylglycerol lipase alpha isoform X1 [Larimichthys crocea] | |
| CH_00047_03215796 | WD repeat-containing protein 3 isoform X1 [Xiphophorus maculatus] | |
| CH_00057_02463358 | Transposable element Tcb2 transposase [Anabarilius grahami] | |
| CH_00057_06026651 | LOW QUALITY PROTEIN: unconventional myosin-IXa-like [Labrus bergylta] | |
| CH_00057_07341171 | PREDICTED: uveal autoantigen with coiled-coil domains and ankyrin repeats [Paralichthys olivaceus] | |
| CH_00079_00193110 | PREDICTED: coagulation factor VII-like, partial [Notothenia coriiceps] | |
| CH_00079_00275405 | PREDICTED: CCR4-NOT transcription complex subunit 8 [Notothenia coriiceps] | |
| CH_00079_00282622 | PREDICTED: gem-associated protein 5 [Notothenia coriiceps] | |
| CH_00079_00305168 | putative 39S ribosomal protein L22 mitochondrial [Scophthalmus maximus] | |
| CH_00084_05899283 | *transcription factor 7 isoform X1 [Perca flavescens] | |
| CH_00084_06081176 | Unknow | |
| CH_00084_06774777 | PREDICTED: rap guanine nucleotide exchange factor 6 isoform X1 [Lates calcarifer] | |
| CH_00086_03578804 | neural proliferation differentiation and control protein 1-like [Perca flavescens]; TDH01950.1 hypothetical protein EPR50_G00168000 [Perca flavescens] | |
| CH_00086_03603366 | PREDICTED: ER degradation-enhancing alpha-mannosidase-like protein 3 [Notothenia coriiceps]; XP_010795726.1 PREDICTED: ER degradation-enhancing alpha-mannosidase-like protein 3 [Notothenia coriiceps] | |
| CH_00086_03619419 | PREDICTED: RNA 3&apos;-terminal phosphate cyclase-like protein [Notothenia coriiceps] | |
| CH_00086_05962124 | LOW QUALITY PROTEIN: nuclear pore complex protein Nup214-like [Labrus bergylta] | |
| CH_00086_05985528 | nucleus accumbens-associated protein 2 [Labrus bergylta]; XP_020494050.1 nucleus accumbens-associated protein 2 [Labrus bergylta]; XP_020494051.1 nucleus accumbens-associated protein 2 [Labrus bergylta]; XP_020494052.1 nucleus accumbens-associated protein 2 [Labrus bergylta] | |
| CH_00089_01093856 | PREDICTED: U4/U6 small nuclear ribonucleoprotein Prp3 [Notothenia coriiceps] | |
| CH_00089_01095148 | Unknow | |
| CH_00089_03828655 | kinesin-like protein KIF13A isoform X6 [Perca flavescens] | |
| CH_00089_06657301 | hypothetical protein EPR50_G00066920 [Perca flavescens] | |
| CH_00112_13947763 | Probable ribosome biogenesis protein RLP24 [Anoplopoma fimbria] | |
| CH_00149_02077462 | *PREDICTED: serum response factor isoform X3 [Notothenia coriiceps] | |
| CH_00163_00902924 | SCAN domain-containing protein 3 [Anabarilius grahami] | |
| CH_00174_02301611 | transient receptor potential cation channel subfamily M member 6-like [Seriola dumerili] | |
| CH_00174_02324066 | nuclear receptor ROR-beta [Labrus bergylta] | |
| CH_00179_02310074 | PREDICTED: phosphoglucomutase-like protein 5 [Notothenia coriiceps] | |
| CH_00179_02414150 | *dedicator of cytokinesis protein 8 isoform X1 [Perca flavescens]; TDH12809.1 hypothetical protein EPR50_G00051830 [Perca flavescens] | |
| CH_00179_02463375 | *PREDICTED: aminopeptidase O-like [Notothenia coriiceps] | |
| CH_00180_02481141 | LOW QUALITY PROTEIN: lipoxygenase homology domain-containing protein 1-like [Acanthochromis polyacanthus] | |
| CH_00180_02695646 | *PREDICTED: SH2B adapter protein 3 [Hippocampus comes] | |
| CH_00190_06192921 | PREDICTED: CUB and sushi domain-containing protein 3-like [Notothenia coriiceps] | |
| CH_00190_06211078 | hypothetical protein EPR50_G00148280 [Perca flavescens] | |
| CH_00193_06822634 | *toll-like receptor 22 [Siniperca chuatsi] | |
| CH_00193_06828505 | PREDICTED: uncharacterized protein LOC104942575, partial [Notothenia coriiceps] | |
| CH_00217_08228042 | PREDICTED: glutamate receptor U1-like [Notothenia coriiceps] | |
| CH_00217_08252192 | PREDICTED: T-cell leukemia translocation-altered gene protein [Notothenia coriiceps] | |
| CH_00217_08254577 | putative glycerate kinase [Scophthalmus maximus] | |
| CH_00217_08258583 | *PREDICTED: WD repeat-containing protein 82 [Notothenia coriiceps] | |
| CH_00217_08265239 | PREDICTED: twinfilin-2 isoform X1 [Notothenia coriiceps] | |
| CH_00249_04715772 | ras-related protein Rab-38 [Oryzias latipes] | |
| CH_00249_04857626 | PREDICTED: cytoplasmic dynein 2 heavy chain 1 isoform X3 [Lates calcarifer] | |
| CH_00282_04814371 | hypothetical protein EPR50_G00116940 [Perca flavescens] | |
| CH_00282_04818739 | PREDICTED: ATP-dependent (S)-NAD(P)H-hydrate dehydratase [Notothenia coriiceps] | |
| CH_00298_01769526 | PREDICTED: receptor-type tyrosine-protein phosphatase delta isoform X1 [Notothenia coriiceps]; XP_010789475.1 PREDICTED: receptor-type tyrosine-protein phosphatase delta isoform X1 [Notothenia coriiceps]; XP_010789476.1 PREDICTED: receptor-type tyrosine-protein phosphatase delta isoform X1 [Notothenia coriiceps] | |
| CH_00298_03598517 | pyruvate carboxylase, mitochondrial isoform X1 [Parambassis ranga] | |
| CH_01361_00028762 | lipoxygenase homology domain-containing protein 1 [Amphiprion ocellaris] | |
| CH_01783_00020832 | Unknow | |
| CH_00217_08236413 | Unknow | |

**Table S13.** The ZD1 specific gene list under selective sweep. (genes with * are discussed in the manuscript)

| Gene ID | annotation |
| --- | --- |
| CH_00001_02186578 | Transposable element Tc1 transposase [Anabarilius grahami] |
| CH_00008_04499033 | PREDICTED: colorectal mutant cancer protein isoform X1 [Notothenia coriiceps] |
| CH_00008_04504608 | PREDICTED: BRISC and BRCA1-A complex member 1 [Stegastes partitus] |
| CH_00008_06035800 | *CREB-regulated transcription coactivator 1 isoform X1 [Larimichthys crocea] |
| CH_00009_04663020 | PREDICTED: neuronal growth regulator 1 isoform X1 [Lates calcarifer] |
| CH_00021_00596192 | PREDICTED: rho GTPase-activating protein 7 isoform X2 [Notothenia coriiceps] |
| CH_00021_03231825 | PREDICTED: uncharacterized protein LOC104968049 [Notothenia coriiceps] |
| CH_00021_03252382 | *rhomboid domain-containing protein 2 [Perca flavescens] |
| CH_00021_03257424 | PREDICTED: OCIA domain-containing protein 1 [Notothenia coriiceps] |
| CH_00022_00501580 | PREDICTED: cysteine protease ATG4D [Notothenia coriiceps] |
| CH_00024_00476542 | zeta-sarcoglycan isoform X1 [Larimichthys crocea] |
| CH_00047_03211222 | sn1-specific diacylglycerol lipase alpha isoform X1 [Larimichthys crocea] |
| CH_00047_03215796 | WD repeat-containing protein 3 isoform X1 [Xiphophorus maculatus] |
| CH_00057_07341171 | PREDICTED: uveal autoantigen with coiled-coil domains and ankyrin repeats [Paralichthys olivaceus] |
| CH_00058_00235679 | PREDICTED: coiled-coil domain-containing protein 81 isoform X1 [Notothenia coriiceps] |
| CH_00084_02106432 | uncharacterized protein LOC109203860 [Oreochromis niloticus] |
| CH_00084_07392930 | PREDICTED: heart- and neural crest derivatives-expressed protein 1 [Notothenia coriiceps] |
| CH_00084_07415363 | PREDICTED: histone deacetylase complex subunit SAP30L isoform X1 [Paralichthys olivaceus] |
| CH_00089_01980125 | putative PIP5K1A and PSMD4-like protein [Phascolarctos cinereus] |
| CH_00089_01984111 | PREDICTED: serum amyloid P-component-like [Notothenia coriiceps] |
| CH_00104_05176419 | *cAMP-responsive element modulator, partial [Ophiophagus hannah] |
| CH_00104_05187260 | PREDICTED: cullin-2 isoform X2 [Notothenia coriiceps] |
| CH_00104_06098607 | ADP-ribosylation factor-like protein 13B [Anabarilius grahami] |
| CH_00112_06168967 | PREDICTED: early endosome antigen 1 isoform X1 [Austrofundulus limnaeus] |
| CH_00121_08463369 | protein unc-13 homolog A [Larimichthys crocea] |
| CH_00134_03131747 | *protein eyes shut homolog [Larimichthys crocea] |
| CH_00134_05325870 | PREDICTED: Hermansky-Pudlak syndrome 1 protein isoform X1 [Notothenia coriiceps] |
| CH_00174_02808294 | PREDICTED: L-serine dehydratase/L-threonine deaminase [Notothenia coriiceps] |
| CH_00179_01479494 | *glutamate receptor ionotropic, delta-2 isoform X1 [Maylandia zebra]; XP_005722070.1 PREDICTED: glutamate receptor ionotropic, delta-2 isoform X1 [Pundamilia nyererei] |
| CH_00179_02242241 | zinc finger SWIM domain-containing protein 6 isoform X1 [Fundulus heteroclitus] |
| CH_00180_01617402 | Unknow |
| CH_00186_00449004 | ADAMTS-like protein 2 [Seriola lalandi dorsalis] |
| CH_00217_10888138 | transmembrane and coiled-coil domains protein 2 [Larimichthys crocea] |
| CH_00217_16013570 | Potassium voltage-gated channel, Shal-related subfamily, member 3 [Dicentrarchus labrax] |
| CH_00226_05569981 | hypothetical protein EPR50_G00232980 [Perca flavescens] |
| CH_00251_02324620 | PREDICTED: protein fosB isoform X1 [Notothenia coriiceps] |
| CH_00282_02921054 | *PREDICTED: growth factor receptor-bound protein 14 isoform X1 [Lates calcarifer] |
| CH_00282_03324578 | LOW QUALITY PROTEIN: protein TANC1 [Perca flavescens] |
| CH_00282_03428313 | death-associated protein-like 1 [Larimichthys crocea] |
| CH_00282_03432988 | PREDICTED: plakophilin-4 isoform X2 [Notothenia coriiceps] |
| CH_00282_03525198 | coiled-coil domain-containing protein 148 isoform X1 [Anabas testudineus] |
| CH_00282_03590884 | hypothetical protein EPR50_G00118770 [Perca flavescens] |
| CH_00282_03602850 | PREDICTED: activin receptor type-1C [Notothenia coriiceps] |
| CH_00298_01619457 | LOW QUALITY PROTEIN: uncharacterized protein LOC106674705 [Maylandia zebra] |
| CH_00298_01769526 | *PREDICTED: receptor-type tyrosine-protein phosphatase delta isoform X1 [Notothenia coriiceps]; XP_010789475.1 PREDICTED: receptor-type tyrosine-protein phosphatase delta isoform X1 [Notothenia coriiceps]; XP_010789476.1 PREDICTED: receptor-type tyrosine-protein phosphatase delta isoform X1 [Notothenia coriiceps] |
| CH_00504_00581727 | PREDICTED: uncharacterized protein LOC104957378 [Notothenia coriiceps] |
| CH_00546_00719407 | *PREDICTED: probable cationic amino acid transporter [Notothenia coriiceps] |
| CH_00546_00725089 | PREDICTED: claudin-11-like [Notothenia coriiceps] |
| CH_01473_00163148 | potassium voltage-gated channel subfamily D member 2-like isoform X2 [Acanthochromis polyacanthus] |

**Table S14** List of sweep regions of RS1, using an outlier approach in RS2. Part of genes with CLR values larger than the genome with 99.9% quantile are shown. Consecutive outlier CLR values are merged to a single sweep region.

| **Rank** | **Pseudo Chromosome** | **Positon (Mbp)** | **MaxCLR** | **Gene with peak** |
| --- | --- | --- | --- | --- |
| 1 | 3 | 2.2 | 9.024183 | CH_01824_00016930 |
| 2 | 3 | 38.2 | 12.874055 | CH_00047_00688122 |
| 3 | 3 | 46.3 | 12.711607 | CH_00349_00425245 |
| 4 | 3 | 46.3 | 12.711607 | CH_01872_00027763, CH_02855_00010213 |
| 5 | 5 | 38 | 6.47159 | CH_00191_00045314 |
| 6 | 5 | 42.4 | 11.817716 | CH_00745_00309884 |
| 7 | 6 | 34.4 | 10.246907 | CH_00139_01446587 |
| 8 | 7 | 9.1 | 9.372744 | CH_00701_00048345, CH_00701_00116347, CH_00701_00274541, CH_00701_00337840, CH_00701_00390318, CH_00868_00107525, CH_04424_00029964 |
| 9 | 8 | 32.6 | 9.576652 | CH_01451_00009773 |
| 10 | 9 | 4.3 | 10.843337 | CH_00184_01099544 |
| 11 | 10 | 34.4 | 9.978282 | CH_00717_00231226 |
| 12 | 13 | 25.3 | 8.797177 | CH_00665_00127650, CH_04763_00000946 |
| 13 | 15 | 25.8 | 5.813969 | CH_00134_00665800 |
| 14 | 16 | 35 | 10.73178 | CH_00926_00004021 |
| 15 | 22 | 16.6 | 11.803463 | CH_00067_00501943 |
| 16 | 22 | 17.5 | 9.062017 | CH_00065_13341480 |
| 17 | 22 | 25.3 | 10.693996 | CH_00065_05518329 |
| 18 | 24 | 24.7 | 9.11694 | CH_01713_00007944 |

**Table S15.** List of sweep regions of RS2, using an outlier approach in RS1. Part of genes with CLR values larger than the genome with 99.9% quantile are shown. Consecutive outlier CLR values are merged to a single sweep region.

| **Rank** | **Pseudo Chromosome** | **Positon (Mbp)** | **MaxCLR** | **Gene with peak** |
| --- | --- | --- | --- | --- |
| 1 | 1 | 24.8 | 25.069327 | CH_00021_01449267 |
| 2 | 1 | 24.8 | 48.283149 | CH_00021_01462569 |
| 3 | 1 | 24.9 | 98.753991 | CH_00021_01552596 |
| 4 | 1 | 24.9 | 83.657848 | CH_00021_01572973 |
| 5 | 1 | 25 | 35.990448 | CH_00021_01623346 |
| 6 | 1 | 25 | 29.068226 | CH_00021_01699753 |
| 7 | 3 | 18.7 | 64.145575 | CH_00057_02463358 |
| 8 | 3 | 22.3 | 86.773364 | CH_00057_06026651 |
| 9 | 3 | 23.6 | 24.24032 | CH_00057_07341171 |
| 10 | 3 | 35.7 | 24.918961 | CH_00047_03211222, CH_00047_03215796 |
| 11 | 4 | 17.1 | 79.958238 | CH_00008_03133903 |
| 12 | 4 | 18.3 | 25.223137 | CH_00008_04327601 |
| 13 | 4 | 18.7 | 37.296688 | CH_00008_04704550 |
| 14 | 4 | 18.7 | 67.828193 | CH_00008_04775876 |
| 15 | 4 | 19 | 66.554506 | CH_00008_04989723, CH_00008_05003079 |
| 16 | 5 | 25.6 | 46.185999 | CH_00217_08228042, CH_00217_08236413, CH_00217_08252192, CH_00217_08254577, CH_00217_08258583, CH_00217_08265239 |
| 17 | 6 | 29 | 72.629898 | CH_00112_13947763 |
| 18 | 9 | 21.5 | 54.058219 | CH_00180_02695646 |
| 19 | 9 | 21.8 | 60.102545 | CH_00180_02481141 |
| 20 | 9 | 21.8 | 87.469415 | CH_01361_00028762 |
| 21 | 9 | 26.6 | 40.72272 | CH_00179_02310074 |
| 22 | 9 | 26.7 | 32.053209 | CH_00179_02414150 |
| 23 | 9 | 26.7 | 32.144238 | CH_00179_02463375 |
| 24 | 9 | 29.6 | 38.282963 | CH_00174_02301611 |
| 25 | 9 | 29.6 | 47.014814 | CH_00174_02324066 |
| 26 | 11 | 16.3 | 56.537546 | CH_00190_06192921, CH_00190_06211078 |
| 27 | 12 | 5.8 | 46.671991 | CH_00086_03578804, CH_00086_03603366, CH_00086_03619419 |
| 28 | 12 | 8.2 | 26.303473 | CH_00086_05962124 |
| 29 | 12 | 8.2 | 42.026144 | CH_00086_05985528 |
| 30 | 13 | 32.1 | 60.128887 | CH_00249_04715772 |
| 31 | 13 | 32.3 | 36.018984 | CH_00249_04857626 |
| 32 | 14 | 9.4 | 25.228248 | CH_00079_00193110 |
| 33 | 14 | 9.5 | 82.495787 | CH_00079_00275405 |
| 34 | 14 | 9.5 | 91.141444 | CH_00079_00282622, CH_00079_00305168 |
| 35 | 14 | 20 | 27.008282 | CH_00084_05899283 |
| 36 | 14 | 20.2 | 40.811918 | CH_00084_06081176 |
| 37 | 14 | 20.9 | 83.231102 | CH_00084_06774777 |
| 38 | 15 | 12.9 | 35.624611 | CH_00149_02077462 |
| 39 | 16 | 9.4 | 46.132151 | CH_00089_01093856, CH_00089_01095148 |
| 40 | 16 | 12.1 | 40.338659 | CH_00089_03828655 |
| 41 | 16 | 14.8 | 26.420664 | CH_01783_00020832 |
| 42 | 16 | 14.9 | 35.204017 | CH_00089_06657301 |
| 43 | 18 | 10.4 | 31.340437 | CH_00298_01769526 |
| 44 | 18 | 12.3 | 24.352568 | CH_00298_03598517 |
| 45 | 19 | 5.4 | 240.61254 | CH_00163_00902924 |
| 46 | 20 | 25.1 | 27.27762 | CH_00193_06822634, CH_00193_06828505 |
| 47 | 21 | 23.5 | 25.493635 | CH_00282_04818739 |
| 48 | 21 | 23.5 | 28.058455 | CH_00282_04814371 |
| 49 | 24 | 12.3 | 70.535985 | CH_00016_01045234,CH_00016_01052259 |

**Table S16.** List of sweep regions of ZD1, using an outlier approach in RS2. Part of genes with CLR values larger than the genome with 99.9% quantile are shown. Consecutive outlier CLR values are merged to a single sweep region.

| **Rank** | **Pseudo Chromosome** | **Positon (Mbp)** | **MaxCLR** | **Gene with peak** |
| --- | --- | --- | --- | --- |
| 1 | 1 | 26.6 | 25.067867 | CH_00021_03252382, CH_00021_03257424 |
| 2 | 1 | 20 | 24.217125 | CH_00024_00476542 |
| 3 | 1 | 23.9 | 20.597824 | CH_00021_00596192 |
| 4 | 1 | 26.6 | 16.208578 | CH_00021_03231825 |
| 5 | 1 | 29.2 | 30.951323 | CH_00022_00501580 |
| 6 | 3 | 23.6 | 16.332855 | CH_00057_07341171 |
| 7 | 3 | 16 | 19.050087 | CH_00058_00235679 |
| 8 | 3 | 35.7 | 20.980755 | CH_00047_03211222, CH_00047_03215796 |
| 9 | 4 | 3 | 15.808428 | CH_00001_02186578 |
| 10 | 4 | 22.8 | 24.746908 | CH_00009_04663020 |
| 11 | 4 | 18.5 | 15.736956 | CH_00008_04499033 |
| 12 | 4 | 18.5 | 11.021174 | CH_00008_04504608 |
| 13 | 4 | 20.1 | 34.571687 | CH_00008_06035800 |
| 14 | 5 | 17.7 | 18.673412 | CH_00217_16013570, CH_01473_00163148 |
| 15 | 5 | 22.6 | 16.742644 | CH_00217_10888138 |
| 16 | 6 | 21.2 | 21.631827 | CH_00112_06168967 |
| 17 | 9 | 22.6 | 11.702407 | CH_00180_01617402 |
| 18 | 9 | 25.8 | 15.423504 | CH_00179_01479494 |
| 19 | 9 | 26.5 | 16.364583 | CH_00179_02242241 |
| 20 | 9 | 30.1 | 77.225589 | CH_00174_02808294 |
| 21 | 9 | 1.1 | 10.757541 | CH_00186_00449004 |
| 22 | 13 | 22.3 | 18.414975 | CH_00251_02324620 |
| 23 | 14 | 16.2 | 30.409003 | CH_00084_02106432 |
| 24 | 14 | 21.5 | 14.518206 | CH_00084_07392930 |
| 25 | 14 | 21.5 | 9.108585 | CH_00084_07415363 |
| 26 | 15 | 1.3 | 9.152519 | CH_00504_00581727 |
| 27 | 15 | 21.1 | 12.854799 | CH_00134_05325870 |
| 28 | 15 | 23.3 | 25.041315 | CH_00134_03131747 |
| 29 | 16 | 10.3 | 19.356881 | CH_00089_01980125, CH_00089_01984111 |
| 30 | 17 | 21.1 | 18.49678 | CH_00121_08463369 |
| 31 | 18 | 10.4 | 25.172722 | CH_00298_01769526, CH_00298_01619457 |
| 32 | 20 | 7.2 | 11.472091 | CH_00104_06098607 |
| 33 | 20 | 8.1 | 21.811576 | CH_00104_05176419, CH_00104_05187260, |
| 34 | 20 | 14.4 | 18.129473 | CH_00546_00719407, CH_00546_00725089 |
| 35 | 21 | 24.7 | 52.333448 | CH_00282_03590884 |
| 36 | 21 | 24.9 | 35.266617 | CH_00282_03428313 |
| 37 | 21 | 25 | 67.854335 | CH_00282_03324578 |
| 38 | 21 | 24.7 | 52.333448 | CH_00282_03602850 |
| 39 | 21 | 24.8 | 20.786797 | CH_00282_03525198 |
| 40 | 21 | 24.9 | 48.952084 | CH_00282_03432988 |
| 41 | 21 | 25.4 | 15.411778 | CH_00282_02921054 |
| 42 | 23 | 21.7 | 12.450476 | CH_00226_05569981 |

**Table S17.** The microbial phyla and classes specifically associated with a *C. hamatus* population.

| **Species** | **Popu-lation** | **Linear discriminant analysis (LDA) value** | ***P* value** |
| --- | --- | --- | --- |
| Patescibacteria.c__Parcubacteria | RS1 | 3.251 | 1.0E-02 |
| Fusobacteriota | RS1 | 2.922 | 3.9E-02 |
| Verrucomicrobiota | RS1 | 3.205 | 1.8E-02 |
| Firmicutes | RS1 | 5.242 | 1.9E-03 |
| Fusobacteriota.c__Fusobacteriia | RS1 | 2.913 | 3.9E-02 |
| Firmicutes.c__Limnochordia | RS1 | 3.607 | 2.7E-02 |
| Patescibacteria.c__Saccharimonadia | RS1 | 3.087 | 2.5E-02 |
| Verrucomicrobiota.c__Chlamydiae | RS1 | 3.198 | 1.8E-02 |
| Acidobacteriota.c__Acidobacteriae | RS1 | 2.924 | 2.1E-02 |
| Firmicutes.c__Bacilli | RS1 | 5.221 | 1.5E-03 |
| Nitrospirota.c__Nitrospiria | RS2 | 3.537 | 5.9E-04 |
| Actinobacteriota | RS2 | 4.476 | 1.6E-02 |
| Desulfobacterota | RS2 | 3.439 | 5.0E-04 |
| Acidobacteriota.c__Holophagae | RS2 | 2.916 | 2.6E-02 |
| Spirochaetota.c__Leptospirae | RS2 | 3.522 | 9.2E-04 |
| Proteobacteria.c__Gammaproteobacteria | RS2 | 5.152 | 2.4E-02 |
| Desulfobacterota.c__norank_p__Desulfobacterota | RS2 | 3.455 | 5.0E-04 |
| Bdellovibrionota | RS2 | 3.064 | 3.2E-02 |
| Actinobacteriota.c__Actinobacteria | RS2 | 4.488 | 1.1E-02 |
| Spirochaetota | RS2 | 3.120 | 2.9E-02 |
| Bdellovibrionota.c__Oligoflexia | RS2 | 3.348 | 2.0E-02 |
| Nitrospirota | RS2 | 3.554 | 5.9E-04 |
| Firmicutes.c__Clostridia | ZD1 | 4.058 | 1.1E-02 |
| Bacteroidota | ZD1 | 3.139 | 3.1E-02 |
| Proteobacteria.c__Alphaproteobacteria | ZD1 | 4.599 | 3.9E-03 |
| Bacteroidota.c__Bacteroidia | ZD1 | 3.139 | 3.1E-02 |
| Proteobacteria | ZD1 | 5.180 | 3.1E-02 |
| Parcubacteria | RS1 | 3.432 | 1.0E-02 |
| Chlamydiae | RS1 | 3.916 | 1.8E-02 |
| Saccharimonadia | RS1 | 3.885 | 2.5E-02 |
| Acidobacteriae | RS1 | 3.135 | 2.1E-02 |
| Limnochordia | RS1 | 4.287 | 2.7E-02 |
| Bacilli | RS1 | 5.494 | 1.5E-03 |
| Fusobacteriia | RS1 | 3.511 | 3.9E-02 |
| Nitrospiria | RS2 | 3.266 | 5.9E-04 |
| norank_p__Desulfobacterota | RS2 | 3.384 | 5.0E-04 |
| Oligoflexia | RS2 | 3.328 | 2.0E-02 |
| Leptospirae | RS2 | 3.402 | 9.2E-04 |
| Holophagae | RS2 | 3.459 | 2.6E-02 |
| Actinobacteria | RS2 | 4.794 | 1.1E-02 |
| Gammaproteobacteria | RS2 | 5.425 | 2.4E-02 |
| Alphaproteobacteria | ZD1 | 4.908 | 3.9E-03 |
| Clostridia | ZD1 | 4.327 | 1.1E-02 |
| Bacteroidia | ZD1 | 3.440 | 3.1E-02 |

**Table S18.** The microbial organisms specifically associated with a *C. hamatus* population.

| **Species** | **Popu-lation** | l**inear discri-minant analysis (LDA) value** | ***P* value** |
| --- | --- | --- | --- |
| Mycoplasma sp. | RS1 | 4.844 | 1.7E-02 |
| Exiguobacterium_sp__ZWU0009 | RS1 | 4.091 | 6.0E-03 |
| unclassified_f__Bacillaceae | RS1 | 4.065 | 2.7E-02 |
| Bacillus thermoamylovorans | RS1 | 3.692 | 2.7E-02 |
| Acinetobacter lwoffii | RS1 | 3.672 | 3.7E-03 |
| Exiguobacterium_sp__AT1b | RS1 | 3.409 | 2.7E-02 |
| uncultured_compost_bacterium_g__norank_f__Limnochordaceae | RS1 | 3.384 | 2.7E-02 |
| Bacteroides_vulgatus | RS1 | 3.380 | 2.5E-02 |
| Trueperella pyogenes | RS1 | 3.310 | 2.7E-02 |
| Blastococcus_aggregatus | RS1 | 3.306 | 2.7E-02 |
| Oceanobacillus sp. | RS1 | 3.158 | 2.7E-02 |
| Pasteurellaceae_bacterium_g__Rodentibacter | RS1 | 3.122 | 2.7E-02 |
| Aeribacillus_pallidus_g__Aeribacillus | RS1 | 3.065 | 2.7E-02 |
| Streptococcus respiraculi | RS1 | 2.977 | 2.7E-02 |
| Bifidobacterium_pseudocatenulatum_DSM_20438__JCM_1200__LMG_10505 | RS1 | 2.924 | 1.2E-03 |
| Williamsia sp. | RS1 | 2.899 | 2.7E-02 |
| Clostridium_baratii_g__Clostridium_sensu_stricto_1 | RS1 | 2.866 | 2.7E-02 |
| uncultured_bacterium_g__Brachybacterium | RS1 | 2.832 | 2.7E-02 |
| Fusobacterium periodonticum | RS1 | 2.739 | 3.9E-02 |
| Bacteroides fragilis | RS1 | 2.600 | 3.0E-02 |
| uncultured_Acidobacteria_bacterium_g__norank_f__norank_o__Subgroup_2 | RS1 | 2.583 | 1.9E-02 |
| unclassified_c__Alphaproteobacteria | RS1 | 2.533 | 3.6E-02 |
| Pseudomonas yamanorum | RS2 | 5.445 | 9.3E-04 |
| Rhodococcus erythropolis | RS2 | 4.867 | 5.3E-04 |
| Ralstonia sp. | RS2 | 4.713 | 9.8E-04 |
| Burkholderia-Caballeronia-Paraburkholderia sp. | RS2 | 4.104 | 6.3E-03 |
| unclassified_g__norank_f__norank_o__norank_c__Alphaproteobacteria | RS2 | 3.557 | 2.8E-04 |
| Leifsonia sp. | RS2 | 3.434 | 1.3E-03 |
| Leptospira sp. | RS2 | 3.419 | 5.7E-04 |
| metagenome_g__Reyranella | RS2 | 3.406 | 1.2E-03 |
| Paraburkholderia ferrariae | RS2 | 3.295 | 1.1E-03 |
| Nitrospira sp. | RS2 | 3.243 | 5.9E-04 |
| unclassified_g__norank_f__norank_o__norank_c__norank_p__Desulfobacterota | RS2 | 3.212 | 5.0E-04 |
| Herbaspirillum_huttiense | RS2 | 3.206 | 2.2E-03 |
| unclassified_g__norank_f__Rhizobiales_Incertae_Sedis | RS2 | 3.121 | 3.9E-02 |
| uncultured_bacterium_g__norank_f__TRA3-20 | RS2 | 3.090 | 1.4E-02 |
| unclassified_g__norank_f__Simkaniaceae | RS2 | 3.048 | 1.2E-03 |
| unclassified_o__Chlamydiales | RS2 | 3.042 | 3.9E-02 |
| unclassified_o__Burkholderiales | RS2 | 3.040 | 3.9E-02 |
| uncultured_bacterium_g__Acidocella | RS2 | 3.023 | 2.8E-04 |
| Thermomonas sp. | RS2 | 3.021 | 2.8E-04 |
| unclassified_c__Gammaproteobacteria | RS2 | 2.950 | 8.8E-03 |
| Pelomonas saccharophila | RS2 | 2.940 | 4.4E-02 |
| Bryobacter sp. | RS2 | 2.936 | 1.4E-02 |
| uncultured_bacterium_g__DSSD61 | RS2 | 2.933 | 3.9E-02 |
| Pedomicrobium sp. | RS2 | 2.910 | 3.9E-02 |
| Paraburkholderia caledonica | RS2 | 2.888 | 3.5E-03 |
| groundwater_metagenome_g__norank_f__norank_o__Candidatus_Kaiserbacteria | RS2 | 2.887 | 1.2E-03 |
| Kosakonia radicincitans | RS2 | 2.862 | 1.8E-02 |
| Mycobacterium mucogenicum | RS2 | 2.859 | 3.9E-03 |
| metagenome_g__MND1 | RS2 | 2.847 | 4.3E-03 |
| uncultured_bacterium_g__norank_f__norank_o__0319-6G20 | RS2 | 2.838 | 4.3E-03 |
| Mesorhizobium sp. | RS2 | 2.832 | 1.2E-03 |
| Gaiella sp. | RS2 | 2.816 | 1.3E-02 |
| uncultured_Acidobacteria_bacterium_g__norank_f__Holophagaceae | RS2 | 2.813 | 2.8E-04 |
| metagenome_g__norank_f__Beijerinckiaceae | RS2 | 2.778 | 1.4E-02 |
| uncultured_beta_proteobacterium_g__norank_f__Neisseriaceae | RS2 | 2.766 | 1.2E-03 |
| uncultured_bacterium_g__Reyranella | RS2 | 2.748 | 2.5E-02 |
| unclassified_g__norank_f__norank_o__Candidatus_Kaiserbacteria | RS2 | 2.707 | 4.3E-03 |
| unclassified_g__norank_f__norank_o__0319-6G20 | RS2 | 2.695 | 3.9E-03 |
| Terrimonas sp. | RS2 | 2.681 | 4.3E-03 |
| Rhodospirillaceae_bacterium_HEG_-6_10 | RS2 | 2.662 | 1.4E-02 |
| uncultured_bacterium_g__Conexibacter | RS2 | 2.434 | 3.9E-02 |
| Ralstonia pickettii | ZD1 | 5.324 | 8.1E-04 |
| Afipia_felis_cat_scratch_disease_bacillus | ZD1 | 4.696 | 3.1E-03 |
| Caulobacter_sp__g__Caulobacter | ZD1 | 4.371 | 3.0E-03 |
| Achromobacter sp. | ZD1 | 4.318 | 1.2E-03 |
| Raoultella sp. | ZD1 | 4.308 | 6.1E-04 |
| Pseudomonas sp. | ZD1 | 4.168 | 3.8E-03 |
| unclassified_g__norank_f__Caulobacteraceae | ZD1 | 4.060 | 3.1E-03 |
| uncultured_Alphaproteobacteria_bacterium_g__Acidisoma | ZD1 | 3.825 | 9.2E-03 |
| Bacillus_anthracis_g__Bacillus | ZD1 | 3.808 | 3.7E-02 |
| Lactococcus_piscium_MKFS47 | ZD1 | 3.655 | 9.2E-03 |
| uncultured_bacterium_g__Methyloversatilis | ZD1 | 3.592 | 1.6E-02 |
| unclassified_o__Enterobacterales | ZD1 | 3.550 | 9.2E-03 |
| Pantoea_ananatis | ZD1 | 3.313 | 5.6E-03 |
| Bosea sp. | ZD1 | 3.162 | 2.6E-02 |
| Yersinia sp. | ZD1 | 3.135 | 9.2E-03 |
| Moraxella_osloensis_g__Enhydrobacter | ZD1 | 3.070 | 3.0E-02 |
| Stenotrophomonas_maltophilia_R551-3 | ZD1 | 3.059 | 1.6E-04 |
| unclassified_g__norank_f__Xanthobacteraceae | ZD1 | 3.051 | 1.8E-02 |
| Pseudomonas_geniculata_g__Stenotrophomonas | ZD1 | 3.044 | 9.2E-03 |
| Serratia sp. | ZD1 | 3.039 | 3.9E-03 |
| Chryseobacterium sp. | ZD1 | 2.919 | 3.8E-02 |
| Massilia sp. | ZD1 | 2.808 | 4.6E-02 |
| Pseudolabrys sp. | ZD1 | 2.659 | 3.2E-02 |

**Table S19.** ZPAX1 and ZPC5 gene copy number tally in eight notothenioids.

| **Species** | **ZPAX1** | **ZPC5** | **Sum** |
| --- | --- | --- | --- |
| *C. gobio* | 8 | 3 | 11 |
| *T. bernacchii* | 13 | 14 | 27 |
| *D. mawsoni* | 15 | 14 | 29 |
| *G. aculeatus* | 29 | 25 | 54 |
| *C. aceratus* | 29 | 31 | 60 |
| *C. hamatus* | 39 | 28 | 67 |
| *P. georgianus* | 27 | 40 | 67 |
| *C. myersi* | 51 | 45 | 96 |

**Table S20.** Data info of seqences, genomes, annotations and reads used in this study.

| **Species** | **Data type** | **Accession No. or URL** | **Database** |
| --- | --- | --- | --- |
| *Chionodraco hamatus* | mitochondrial genome seq. | NC_029737.1 | GenBank |
| *Chionodraco myersi* | mitochondrial genome seq. | NC_010689.1 | GenBank |
| *Chionodraco myersi* | genome Illumina reads | SRX5016434 | GenBank |
| [*Chionodraco rastrospinosus*](https://www.ncbi.nlm.nih.gov/Taxonomy/Browser/wwwtax.cgi?id=34790) | mitochondrial genome seq. | NC_039543.1 | GenBank |
| *Chionodraco hamatus* | Trim35 complete cds. | OM337937.1 | GenBank |
| *Chionodraco hamatus* | Trim39-like complete cds. | OM337938.1 | GenBank |
| *Larimichthys crocea* | genome seq. and annotations | https://doi.org/10.6084/m9.figshare.c.4312241.v1 | figshare |
| *Dissostichus Mawsoni* | genome seq. and annotations | http://202.121.66.128/ | ocean genome database |
| *Eleginops maclovinus* | genome seq. and annotations | http://202.121.66.128/ | ocean genome database |
| *Chaenocephalus aceratus* | genome seq. and annotations | https://antagen.kopri.re.kr/project/genome_info_ifra me.php?Code=CA00 | AntaGen |
| *Parachaenichthys charcoti* | genome seq. and annotations | http://gigadb.org/dataset/100321# | GigaDB |
| *Gasterosteus aculeatus* | genome seq. and annotations | GCA_016920845.1 | Ensembl |
| *Oryzias latipes* | genome seq. and annotations | GCA_002234715.1 | Ensembl |
| *Oreochromis niloticus* | genome seq. and annotations | GCA_001858045.3 | Ensembl |
| *Cottoperca gobio* | genome seq. and annotations | GCA_900634415.1 | GenBank |
| *Chionodraco myersi* | genome seq. and annotations | GCA_009756435.1 | GenBank |
| *Notothenia coriiceps* | genome seq. and annotations | GCA_000735185.1 | GenBank |
| *Perca flavescens* | genome seq. and annotations | GCA_004354835.1 | GenBank |
| *Pseudochaenichthys georgianus* | genome seq. and annotations | GCA_902827115.1 | GenBank |
| *Trematomus bernacchii* | genome seq. and annotations | GCF_902827165.1 | GenBank |
